# Biomimetic thiyl radical formation from diphenyl disulfide with the low valent Ni(i) state of a cofactor F430 model†

**DOI:** 10.1039/d4sc08416k

**Published:** 2025-01-20

**Authors:** Samira Amini, Kerstin Oppelt, Olivier Blacque, Mikhail Agrachev, Gunnar Jeschke, Felix Zelder

**Affiliations:** a Department of Chemistry, University of Zurich Winterthurerstrasse 190 CH-8057 Zurich Switzerland felix.zelder@chem.uzh.ch +41 44 635 6803; b Institute of Molecular Physical Science, ETH Zurich Vladimir-Prelog-Weg 2 CH-8093 Zurich Switzerland

## Abstract

Cofactor F430 is a nickel-containing hydrocorphinato complex that plays important roles in the enzymatic formation and oxidation of methane. In methanotrophic bacteria, F430-dependent methyl-coenzyme M reductase (MCR) catalyses the endergonic conversion of the heterodisulfide adduct of coenzymes M and B with methane to methyl-coenzyme M and coenzyme B. In a radical mechanism, the Ni(i)-induced formation of a transient thiyl radical of coenzyme B from the heterodisulfide has been proposed. Herein, we introduce a new semi-artificial Ni-complex derived from vitamin B_12_ as functional model of F430. We demonstrate with electrochemical studies that the low valent Ni(i) complex cleaves the biomimetic model compound diphenyl disulfide into approx. 0.5 equivalents of thiophenol and a transient thiophenyl radical at a potential of −1.65 V *vs.* Fc/Fc^+^. Thiyl radicals are trapped in solution with phenylacetylene as thiophenyl-substituted olefins, but also lead to degradation of the Ni-complex.

## Abstract 2

The low valent Ni(i) state of a new cofactor F430 model cleaves a biomimetic disulfide to a thiol and a transient thiyl radical as proposed for anaerobic oxidation of methane with F430-dependent methyl-coenzyme M reductase (MCR).

## Graphical abstract

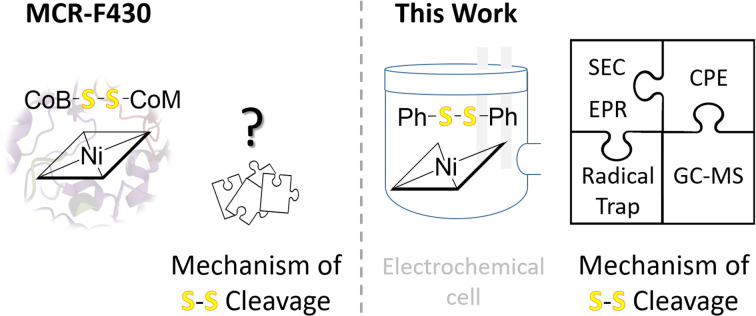

## Introduction

Anaerobic oxidation of methane (AOM) plays an important role in the global carbon cycle.^1,2^ In this way, a hundred million tons of CH_4_ are oxidized annually to CO_2_ by the interplay of methanotrophic archaea and sulfate-reducing bacteria in marine sediments (eqn (1)).^3^ This biotransformation prevents the release of large amounts of the highly potent greenhouse gas methane into the atmosphere.^4^

1 CH_4_ + SO_4_^2−^ → HCO_3_^−^ + HS^−^ + H_2_O, Δ*G*°′ = −17 kJ mol^−1^

**Fig. 1:**
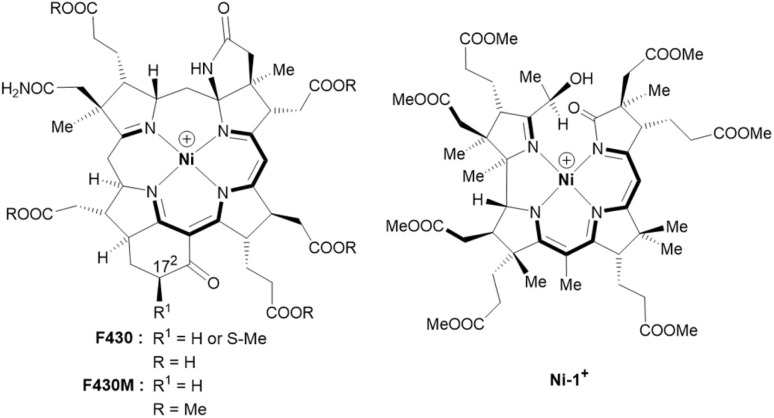
Structural formulas and core structures of cofactor F430 and F430M (left) and of the new seconibester model Ni-1^+^ (right). The (10 + 2) π-electron core structures are shown in bold.

**Scheme 1:**
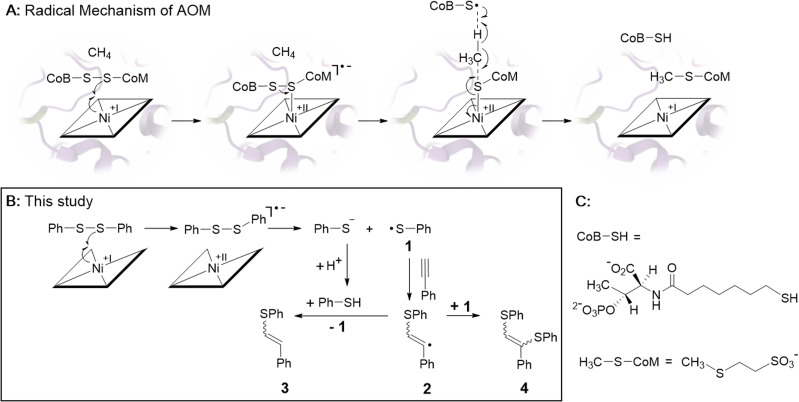
(A) Proposed radical mechanism of AOM catalyzed by F430-dependent MCR (ref. 1 and 2). (B) Biomimetic electrochemistry study. Electrogenerated Ni-1 reduces diphenyl disulfide (PhSSPh) to thiophenol (PhSH) and thiophenyl radical 1. Radical 1 adds to phenylacetylene yielding vinyl-radical 2 that is further converted to olefins 3 and 4 (ref. 28). (C) Structures of CoB-SH and H_3_C-SCoM.

In 2003, the groups of Shima and Thauer as well as of deLong independently suggested methyl-coenzyme M reductase (MCR) as the key enzyme of AOM.^5,6^ MCR depends on cofactor F430, a tetradentate Ni-hydrocorphin complex with either a hydrogen or a *S*-configured methylthio group at position 17^2^ of the macrocyclic ligand (Fig. 1, left).^7^ The low valent Ni(i) state of F430 is critical for the enzymatic formation of methyl-coenzyme M (H_3_C–S-CoM, Scheme 1C) and coenzyme B (HS-CoB, Scheme 1C) from CH_4_ and the heterodisulfide adduct of coenzyme M and coenzyme B (CoM–S–S–CoB; eqn (2)).^8,9^

This endergonic reaction (Δ*G*° = 30 kJ mol^−1^) proceeds as the reverse of exergonic methane formation (Δ*G*° = −30 kJ mol^−1^) in the metabolism of chemotrophic methanogens (eqn (2) from right to left).^1^ Since methane oxidation and methane formation are catalyzed by the same F430-dependent enzyme (*i.e.* MCR),^10–12^ the forward and back reactions described by eqn (2) should proceed *via* the same mechanism according to the concept of microscopic reversibility.^13^

2 CH_4_ + CoM–S–S–CoB ⇌ CH_3_–S–CoM + HS-CoB, Δ*G*° = +30 ± 10 kJ mol^−1^

Among different mechanistic proposals,^14–17^ a Ni(i)-induced radical cascade reaction has been suggested by computational studies and was later supported by transient kinetic spectroscopy studies.^2,16,18^ In this mechanism, the low valent Ni(i) form of F430 converts the heterodisulfide CoM–S–S–CoB in a one-electron reduction to Ni(ii)–S–CoM and a transient thiyl-radical of coenzyme B (Scheme 1A). The latter abstracts a hydrogen atom from methane, which is converted into H_3_C–S–CoM (Schemes 1A and C). The endergonic homolytic scission of the particularly strong C–H bond of methane (439 kJ mol^−1^) with a thiyl radical is unknown in synthetic chemistry, but has precedence in other enzymatic systems.^19^ It probably proceeds through a concerted mechanism as suggested earlier for anaerobic hexane activation by a thiyl radical.^20^

Fundamental work on properties and reactivity of F430 in apolar solvents was reported by Jaun and colleagues with F430M (Fig. 1, left), a non-polar pentamethyl ester model of native F430 mimicking the hydrophobic active site of MCR.^21,22^ In contrast to these fundamental studies, biomimetic AOM chemistry has not been studied so far with F_430_ models.^23–27^

With this *terra incognita* in mind, we herein describe a new functional model of F430 in the low valent Ni(i) state that induces transient thiyl radical formation by reductive cleavage of a model disulfide.

The semi-artificial Ni-complex Ni-1^+^ (Fig. 1, right) contains a central Ni(ii)-ion embedded in a tetradentate seconibester ligand derived from vitamin B_12_.

The pseudo-macrocyclic (10 + 2) π-electron system is reminiscent to the electronic core structure of F430 and contains additional electron-withdrawing keto and hydroxy groups similar to modifications at the periphery of its natural counterpart F430 (Fig. 1). Structurally different seconirrin derivatives have been developed earlier in the group of Albert Eschenmoser during the two total syntheses of vitamin B_12_, but were not investigated in biomimetic and electrocatalytic studies.^21,29–31^

## Experimental

**Fig. 2:**
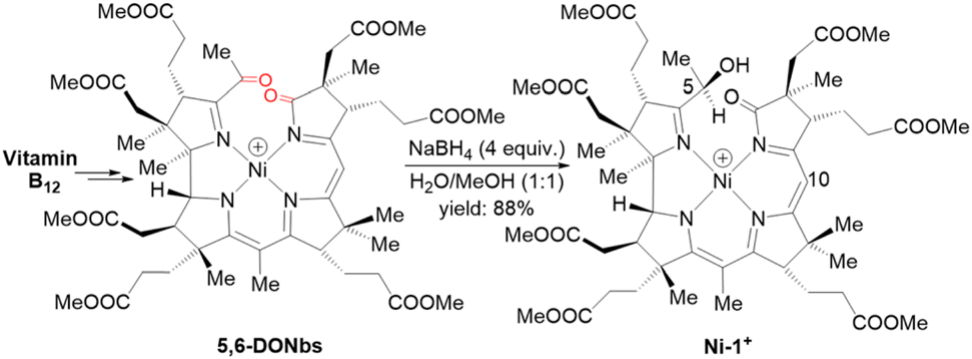
Synthetic route towards the F430 model Ni-1^+^ starting from vitamin B_12_. Only the last step is shown (positions at C5 and C10 are indicated).

For the preparation of Ni-1^+^, we first synthesized 5,6-dioxo-5,6-*seco*-heptamethyl nibyrinate (5,6-DONbs) from vitamin B_12_ in an isolated yield of 33% according to a procedure developed earlier in the Zelder group.^24,32,33^ Subsequently, 5,6-DONbs was reduced with NaBH_4_ to the new 5-hydroxy-6-oxo-5,6-*seco*-heptamethylnibyrinate (Ni-1^+^) and isolated as ClO_4_^−^ salt (88%, Fig. 2).

## Results and discussion

The reduction of the keto-group of 5,6-DONbs to a hydroxy functionality was pursued to prevent undesired ligand-centered redox chemistry during electrochemical studies. The structural integrity of Ni-1^+^ was verified by HR-ESI-MS (M^+^, *m*/*z*_exp_: 1069.45084; M^+^, *m*/*z*_calc_: 1069.45261 for C_52_H_75_N_4_NiO_16_^+^) and spectroscopic investigations using UV/vis, as well as homo- and heteronuclear one- and two-dimensional NMR spectroscopy (Fig. S2–S9†). The visible region of the absorption spectrum in CH_3_CN is reminiscent to the spectrum of hydrophobic F430M (Fig. 1 left) with a red shifted maximum at 461 nm (Δ*λ* = 30 nm; log *ε* = 3.94) (Fig. S2†).^22^ The NMR spectra of Ni-1^+^ showed sharp peaks indicating a square-planar coordination geometry of Ni(ii) with a d^8^-configuration. The HMBC spectrum lacked correlations between the protons of the methyl group at C51 (1.81 ppm) and C6 (189.27 ppm) as expected for a pseudo-macrocyclic seconirrin ligand (Fig. S1 and S9†). Although, we were not able to grow suitable crystals for structural analysis, the configuration was tentatively assigned by ^1^H–^1^H NOESY spectroscopy on the DFT-optimized geometries. These data revealed an *R* configuration on C5 (Table S2†).

**Fig. 3:**
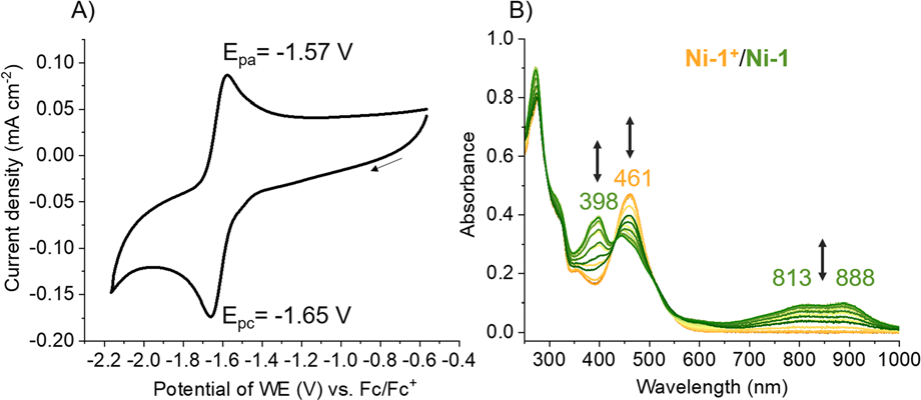
(A) Cyclic voltammogram of Ni-1^+^ (1.5 mM) recorded at 0.1 V s^−1^ in CH_3_CN containing TBAPF_6_ (0.1 M). (B) UV/vis-SEC of Ni-1^+^ (2.65 mM) recorded in CH_3_CN containing TBAPF_6_ (0.6 M). Ni-1^+^ contains a Ni(ii)-ion whereas Ni-1 contains a Ni(i)-ion.

Cyclic voltammetry (CV) studies of Ni-1^+^ in CH_3_CN showed a quasi-reversible redox feature (*E*_1/2_ = −1.61 V; Δ*E*_p_ = 80 mV) with a distinct cathodic wave at *E*_pc_ = – 1.65 V *vs.* Fc/Fc^+^ (Fig. 3A). The observed *E*_1/2_-value is more negative to the one of F430M in DMF (*E*_1/2_ = −1.32 V *vs.* Fc/Fc^+^).^22^ The reduction of Ni-1^+^ (3.7 mM in MeCN) was additionally studied with spectroelectrochemical (SEC) methods in an OTTLE cell exhibiting a reversible reduction wave at −1.81 V *vs.* Fc/Fc^+^ (Fig. S11 and Table S3†). At potentials more negative than −1.4 V *vs.* Fc/Fc^+^, changes in the UV/vis spectra of Ni-1^+^ were observed with isosbestic points at 327 and 430 nm indicating a clean conversion to a single product with a strong absorption at 382 nm and additional characteristic weaker bands in the NIR region at 813 and 888 nm. This SEC behavior shows remarkable similarities with that of F430(M) upon one-electron metal-centered reduction.^18,22^ For the natural cofactor F430 and its hydrophobic derivative F430M (Fig. 1, left), changes in the NIR region have been ascribed to a metal-to-ligand-charge transfer from Ni(i) to the hydrocorphinato ligand (Fig. 3B).^34^

Additional evidence for a metal-centered reduction was obtained from continuous wave (CW) EPR measurements of Ni-1 in frozen toluene/MeCN at 10 K (Fig. S12†). The spectrum consists of two main features with a broader signal A centered at lower fields and a narrow signal B. Double integration of the simulated signals indicates approximately 95% of species A and only 5% of species B. The main anisotropic component A, with principal **g** values (*g*_‖_ = 2.175, *g*_⊥_ = 1.980) with sizeable differences from *g*_e_, is attributed to a metal-localized paramagnetic center, and specifically to a Ni(i) species (d^9^, *S* = 1/2). The spectrum resembles those of square-planar hydrophobic F430M (*g*_‖_ = 2.250, *g*_⊥_ = 2.065)^22^ and, in particular, a previously reported Ni-corrin derivative (*g*_‖_ = 2.194, *g*_⊥_ = 1.980).^24^ For the minor component B, the narrow linewidth, low **g** anisotropy and small deviation of the average *g* value (*g*_‖_ = 1.997, *g*_⊥_ = 2.005) from the free electron *g*_e_ ≈ 2.0023 suggests a ligand-centered radical.

**Fig. 4:**
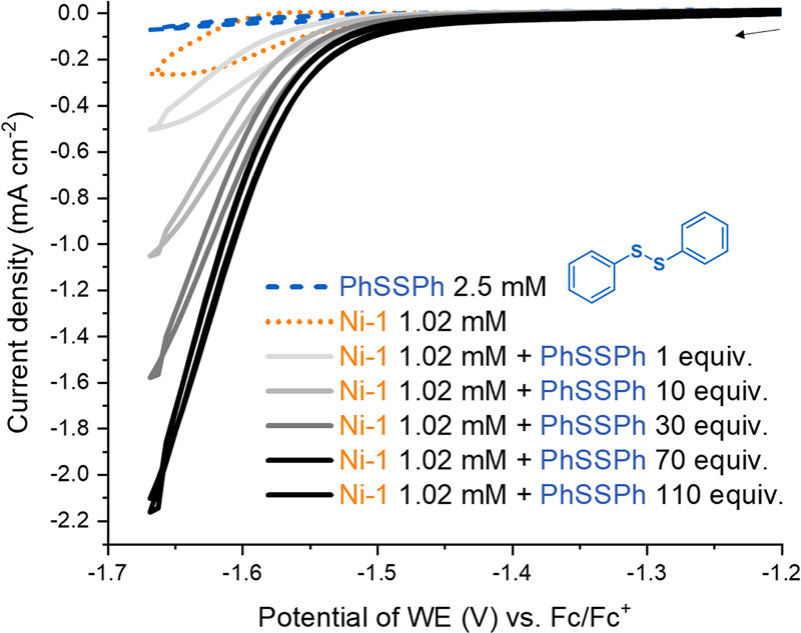
Cyclic voltammogram (CV) of diphenyl disulfide (PhSSPh, 2.5 mM, blue dashed) and of Ni-1[ClO_4_] (1.02 mM, orange dotted) in the absence and presence of diphenyl disulfide (1, 10, 30, 70, 110 equiv., gray and black solid) at 0.1 V s^−1^ in CH_3_CN containing TBAPF_6_ (0.1 M). CV scans were initiated at −1.16 V *vs.* Fc/Fc^+^.

In biomimetic electrochemistry studies, we tested low valent Ni-1 for the reductive cleavage of different model disulfides as proposed in the first step of the mechanism of AOM (Scheme 1A). Cystine and dibenzyl disulfide were initially considered as models of the aliphatic heterodisulfide CoM-S-S-CoB, but cystine showed poor solubility in CH_3_CN, whereas the redox potential difference between dibenzyl disulfide (*E*_pc_ = – 2.72 V *vs.* Fc/Fc^+^) and Ni-1^+^ (*E*_pc_ = – 1.65 V *vs.* Fc/Fc^+^) was too large for successful electrocatalysis.^35^ In contrast to the inertness of Ni-1 toward dibenzyl disulfide, MCR-bound F430 cleaves the aliphatic archetype CoM-S-S-CoB, although F430(M) exhibits a more positive reduction potential than Ni-1^+^.^22,36^ We propose that protein binding alters both the inner-,^37^ and secondary coordination sphere of F430, shifting its reduction potential to more negative values, thereby enabling reductive cleavage.^9^ The impact of the secondary coordination sphere on the reduction potential of a Ni(ii)-complex has already been demonstrated in a related protein-F430 model.^27^ However, the reduction potential of MCR-bound F430 has not been reported so far.^13^ In contrast to cystine and dibenzyl disulfide, diphenyl disulfide (PhSSPh, Scheme 1B) exhibits a more positive standard reduction potential (*E*_pc_ = −2.09 V *vs.* Fc/Fc^+^).^35^ Addition of PhSSPh in CV experiments with Ni-1^+^ resulted in a catalytic current attributed to substrate reduction with an onset at −1.65 V *vs.* Fc/Fc^+^ that follows reduction of Ni-1^+^ to Ni-1 (Fig. 4). The CV became irreversible and the catalytic current increased with increasing concentration of the substrate (0–110 equiv.; Fig. 4). In the absence of the Ni-complex reduction of PhSSPh was only observed at significantly more negative potentials (Δ*E* = – 0.443 V; Fig. S13†).^35^

Controlled potential electrolysis (CPE) of PhSSPh (20 mM; 20 equiv.) was performed in CH_3_CN/water (16% v/v) and TBAPF_6_ (0.1 M) at potentials of −1.65 V *vs.* Fc/Fc^+^ for 90 min in the presence and absence of Ni-1 (1 equiv.). Whereas no cleavage of PhSSPh to PhSH was observed in the absence of Ni-1 (Fig. S14†), approximately 0.5 equiv. of PhSH (0.55 mM) were detected in the presence of Ni-1 based on GC-MS analyses (Fig. S15 and S16†). LC-UV/vis analysis of the reaction mixture revealed degradation of Ni-1 (Fig. S17†) into different products as undesired side reaction. Since Ni-1 was not affected by the presence of PhSH in control experiments, we speculate that the transient thiyl radical PhS˙ (1, Scheme 1B) triggers degradation of Ni-1. A similar behaviour has been observed earlier for MCR upon reduction in the presence of the heterodisulfide CoM-S-S-CoB.^38^ The resulting inactivation of the enzyme has been explained by uncontrolled reactions of the thiyl-radical of coenzyme B derived from the Ni(i)-induced cleavage of the heterodisulfide.^13^ For this reason, we attempted to catch the reactive thiyl-radical 1 with a radical trap. Phenylacetylene seemed perfectly suited,^28,39^ because the addition of thiyl-radicals to alkynes is well explored and terminal alkynes are largely unreactive towards nucleophilic additions of thiols.^40,41^ In these CPE experiments, we observed formation of the thiyl-radical adducts 3 and 4 (Scheme 1B) with GC-MS analysis (Fig. S18,†*R*_t_ = 12.63 and 17.37 min) in the presence, but not the absence of the Ni-complex (Fig. S19†). In accordance with a mechanism proposed by Ishibashi and co-workers (Scheme 1B),^28^ we suggest the formation of PhSH and the transient thiyl radical 1 upon Ni(i) induced cleavage of PhSSPh. The subsequent addition of the thiyl species to phenylacetylene yields vinyl radical 2 that either abstracts a hydrogen atom of PhSH to yield 3 or reacts with 1 to form 4.

Subsequently, we also tested the hydrogen atom abstraction from cumene (BDE = 84.5 kcal mol^−1^)^42^ as an activated model of methane in our CPE experiments with Ni-1^+^ and PhSSPh. Neither the formation of dicumene or any other cumene derivative was observed in the reaction mixture (Fig. S20†) hinting towards radical C–H activation of the substrate.^43^ The RS–H bond dissociation energy (BDE) of thiophenol (PhSH; BDE = 79 kcal)^39^ is probably too low for hydrogen atom transfer from cumene to 1.

## Conclusions

In summary, we present a semi-artificial Ni-complex derived from vitamin B_12_ as structural and functional model of cofactor F430. The low-valent Ni(i) state of the biomimetic complex exhibits striking spectroelectrochemical similarities with its natural archetype and exhibits a half wave potential (*E*_1/2_) of −1.61 V *vs.* Fc/Fc^+^. This strong reducing power of the Ni(i) state makes the one-electron cleavage of diphenyl disulfide into approx. 0.5 equivalents of thiophenol and a thiophenyl radical possible. The transient thiyl radical was trapped with phenylacetylene as thiophenyl substituted olefins 3 and 4, but leads also to degradation of the Ni-complex. The activation of C–H bonds in cumene was attempted, but not observed due to the low BDE of thiophenol (BDE = 79 kcal mol^−1^). For achieving radical C–H activation in the future, the cleavage of disulfides with a significantly higher BDE and a more negative reduction potential than thiophenol is required. For this purpose, the incorporation of Ni-1^+^ into protein-models to explore additional secondary coordination sphere effects is suggested. In addition, our group develops new Ni-corrin derivatives with a more negative reduction potential than Ni-1^+^.

Overall, the present electrochemical model reaction mimics the first step of the radical mechanism in the anaerobic oxidation of methane with MCR and contributes therefore to the current understanding of F430-catalyzed reactions.

## Data availability

The data supporting this article have been included as part of the ESI.†

## Author contributions

F. Zelder and S. Amini designed experiments and wrote the manuscript. Samira Amini conducted all experiments despite EPR-, and spectroelectrochemical studies. K. Oppelt performed spectroelectrochemical studies and assisted in the writing of the manuscript. M. Agrachev performed EPR measurements, analysed the results with G. Jeschke and assisted in the writing of the manuscript. Dr Olivier Blaque provided DFT calculation and assisted in the writing of the manuscript.

## Conflicts of interest

There are no conflicts to declare.

## Supplementary Material

SC-OLF-D4SC08416K-s001

## References

[cit1] Thauer R. K. (2010). Angew. Chem., Int. Ed..

[cit2] Wongnate T., Sliwa D., Ginovska B., Smith D., Wolf M. W., Lehnert N., Raugei S., Ragsdale S. W. (2016). Science.

[cit3] Shima S., Krueger M., Weinert T., Demmer U., Kahnt J., Thauer R. K., Ermler U. (2012). Nature.

[cit4] Reeburgh W. S. (2007). Chem. Rev..

[cit5] Krüger M., Meyerdierks A., Glöckner F. O., Amann R., Widdel F., Kube M., Reinhardt R., Kahnt R., Böcher R., Thauer R. K., Shima S. (2003). Nature.

[cit6] Hallam S. J., Girguis P. R., Preston C. M., Richardson P. M., DeLong E. F. (2003). Appl. Environ. Microbiol..

[cit7] Mayr S., Latkoczy C., Kruger M., Gunther D., Shima S., Thauer R. K., Widdel F., Jaun B. (2008). J. Am. Chem. Soc..

[cit8] Pfaltz A., Jaun B., Fässler A., Eschenmoser A., Jaenchen R., Gilles H. H., Diekert G., Thauer R. K. (1982). Helv. Chim. Acta.

[cit9] Scheller S., Goenrich M., Boecher R., Thauer R. K., Jaun B. (2010). Nature.

[cit10] Goubeaud M., Schreiner G., Thauer R. K. (1997). Eur. J. Biochem..

[cit11] Shima S., Goubeaud M., Vinzenz D., Thauer R. K., Ermler U. (1997). J. Biochem..

[cit12] Mcbride B. C., Wolfe R. S. (1971). Biochemistry.

[cit13] Thauer R. K. (2019). Biochemistry.

[cit14] Ermler U., Grabarse W., Shima S., Goubeaud M., Thauer R. K. (1997). Science.

[cit15] Jaun B., Pfaltz A. (1988). J. Chem. Soc., Chem. Commun..

[cit16] Chen S. L., Blomberg M. R. A., Siegbahn P. E. M. (2012). Chem.–Eur. J..

[cit17] Scheller S., Goenrich M., Mayr S., Thauer R. K., Jaun B. (2010). Angew. Chem., Int. Ed..

[cit18] Patwardhan A., Sarangi R., Ginovska B., Raugei S., Ragsdale S. W. (2021). J. Am. Chem. Soc..

[cit19] Rabus R., Boll M., Heider J., Meckenstock R. U., Buckel W., Einsle O., Ermler U., Golding B. T., Gunsalus R. P., Kroneck P. M. H., Krüger M., Lueders T., Martins B. M., Musat F., Richnow H. H., Schink B., Seifert J., Szaleniec M., Treude T., Ullmann G. M., Vogt C., von Bergen M., Wilkes H. (2016). J. Mol. Microbiol. Biotechnol..

[cit20] Jarling R., Sadeghi M., Drozdowska M., Lahme S., Buckel W., Rabus R., Widdel F., Golding B. T., Wilkes H. (2012). Angew. Chem., Int. Ed..

[cit21] Kratky C., Fässler A., Pfaltz A., Kräutler B., Jaun B., Eschenmoser A. (1984). J. Chem. Soc., Chem. Commun..

[cit22] Jaun B., Pfaltz A. (1986). J. Chem. Soc. Chem. Commun..

[cit23] Miyazaki Y., Oohora K., Hayashi T. (2022). Chem. Soc. Rev..

[cit24] Brenig C., Mosberger L., Blacque O., Kissner R., Zelder F. (2021). Chem. Commun..

[cit25] Kieninger C., Wurst K., Podewitz M., Stanley M., Deery E., Lawrence A. D., Liedl K. R., Warren M. J., Kräutler B. (2020). Angew. Chem., Int. Ed..

[cit26] Ghosh A., Conradie J. (2023). J. Inorg. Biochem..

[cit27] Oohora K., Miyazaki Y., Hayashi T. (2019). Angew. Chem., Int. Ed..

[cit28] Taniguchi T., Fujii T., Idota A., Ishibashi H. (2009). Org. Lett..

[cit29] Eschenmoser A. (1988). Angew. Chem., Int. Ed..

[cit30] Yamada Y., Miljkovic D., Wehrli P., Golding B., Loliger P., Keese R., Muller K., Eschenmoser A. (1969). Angew. Chem., Int. Ed..

[cit31] Fässler A., Pfaltz A., Müller P. M., Farooq S., Kratky C., Kräutler B., Eschenmoser A. (1982). Helv. Chim. Acta.

[cit32] Brenig C., Prieto L., Oetterli R., Zelder F. (2018). Angew. Chem., Int. Ed..

[cit33] Zelder F. H., Buchwalder C., Oetterli R. M., Alberto R. (2010). Chem.–Eur. J..

[cit34] Craft J. L., Horng Y. C., Ragsdale S. W., Brunold T. C. (2004). J. Am. Chem. Soc..

[cit35] Zhu Q. L., Costentin C., Stubbe J., Nocera D. G. (2023). Chem. Sci..

[cit36] Holliger C., Pierik A. J., Reijerse E. J., Hagen W. R. (1993). J. Am. Chem. Soc..

[cit37] F430M exhibits in acetonitrile the coordination number 4, whereas MCR-bound F430 exhibits coordination numbers 5 or 6

[cit38] Prakash D., Wu Y. N., Suh S. J., Duin E. C. (2014). J. Bacteriol..

[cit39] Dénès F., Pichowicz M., Povie G., Renaud P. (2014). Chem. Rev..

[cit40] Oswald A. A., Griesbaum K., Bregman J. M., Hudson B. E. (1964). J. Am. Chem. Soc..

[cit41] Koeckenberger J., Klemt I., Sauer C., Arkhypov A., Reshetnikov V., Mokhir A., Heinrich M. R. (2023). Chem.–Eur. J..

[cit42] Ghosh M., Singh K. K., Panda C., Weitz A., Hendrich M. P., Collins T. J., Dhar B. B., Sen Gupta S. (2014). J. Am. Chem. Soc..

[cit43] Walling C., Rabinowitz R. (1959). J. Am. Chem. Soc..

