# Supplementary material for: Biomimetic thiyl radical formation from diphenyl disulfide with the low valent Ni(i) state of a cofactor F430 model
Source: Chem Sci. 2025 Jan 20;16(10):4290–4. doi: 10.1039/d4sc08416k (PMC11791513; doi:10.1039/d4sc08416k)
Supplement: SC-016-D4SC08416K-s001 [file SC-016-D4SC08416K-s001.pdf]

Supplementary Information (SI) for Chemical Science.
This journal is © The Royal Society of Chemistry 2025

Supporting Information

Biomimetic Thiyl Radical Formation From Diphenyldisulphide with The Low Valent Ni(I) State of A Cofactor F430 Model

Samira Amini,[a] Dr. Kerstin Oppelt,[a] Dr. Olivier Blacque,[a] Dr. Mikhail Agrachev,[b] Prof. Dr. Gunnar Jeschke[b] and Prof. Dr. Felix Zelder[a]

[a]Department of Chemistry, University of Zurich, Winterthurerstrasse 190, CH-8057 Zurich, Switzerland. Fax: +41 44 635 6803; E-mail: felix.zelder@chem.uzh.ch

[b]Institute of Molecular Physical Science, ETH Zurich, Vladimir-Prelog-Weg 2, CH-8093 Zurich, Switzerland

Contents

Materials and Methods....................S3
Reaction Schemes....................S7
Scheme S1. Synthesis of **DCCbs**.[S1,S2] S7
Scheme S2. Photo-oxygenolysis of **DCCbs** to generate **5,6-DODCCbs**.[S3] S8
Scheme S3. Synthesis of **5,6-DONibs[ClO$_4$]** from **5,6-DODCCbs**.[S4] S8
Scheme S4. Synthesis of **Ni-1[ClO$_4$]** from **5,6-DONibs[ClO$_4$]**. S9
Experimental Procedure: S9
Co$_{\alpha\beta}$-dicyano heptamethyl cobyrinate (**DCCbs**): S9
5,6-dioxo-5,6-*seco* Co$_{\alpha\beta}$-dicyano heptamethyl cobyrinate (**5,6-DODCCbs**): S9
5,6-dioxo-5,6-*seco*-heptamethyl nibyrinate perchlorate (**5,6-DONibs[ClO$_4$]**): S10
(5R)-5-hydroxy-6-oxo-5,6-*seco*-heptamethyl nibyrinate perchlorate (**Ni-1[ClO$_4$]**): S10
Atom Numbering of **Ni-1[ClO$_4$]**: S11
Figure S1. Atom Numbering of **Ni-1[ClO$_4$]** S11
Analytical Data: S12
(5R)-5-hydroxy-6-oxo-5,6-*seco*-heptamethyl nibyrinate perchlorate (**Ni-1[ClO$_4$]**): S12
Figure S2. UV/Vis spectrum of **Ni-1[ClO$_4$]** S12
Figure S3. (+)-HR-ESI-MS of **Ni-1[ClO$_4$]**....................S12
Figure S4. ^{1}H-NMR of **Ni-1[ClO$_4$]**. S13
Figure S5. ^{13}C-NMR of **Ni-1[ClO$_4$]**. S13
Figure S6. COSY of **Ni-1[ClO$_4$]**. S14
Figure S7. NOESY of **Ni-1[ClO$_4$]**. S14
Figure S8. HSQC of **Ni-1[ClO4]**. S15
Figure S9. HMBC of **Ni-1[ClO$_4$]**. S15
Table S1. ^{1}H-NMR chemical shifts and ^{13}C-NMR chemical shifts of **Ni-1[ClO$_4$]**. S16
Table S2. Determination of C$_5$ configuration in **Ni-1[ClO$_4$]** S17
Electrochemistry and Analysis: S17
Table S3. Overview of electrodes and cells used in electrochemical studies. S17
Figure S10. Cyclic voltammograms of **Ni-1[ClO$_4$]** S18
Figure S11. Cyclic voltammograms of **Ni-1[ClO$_4$]** in OTTLE cell S18
Figure S12. CW-EPR spectrum of **Ni-1**. S19
Figure S13. Cyclic voltammograms of diphenyldisulphide (**PhSSPh**) S19
Figure S14. GC-MS chromatogram of the CPE experiment with **PhSSPh** without **Ni-1[ClO$_4$]** S20

Figure S15. GC-MS chromatogram of the CPE experiment with **PhSSPh** and **Ni-1[ClO$_4$]** .S21
Figure S16. Calibration curve and quantification of **[PhSH]**$_{sample}$ S22
Figure S17. Chromatogram of **Ni-1[ClO$_4$]** before and after CPE S23
Figure S18. GC-MS of the CPE experiment of **PhSSPh** with **Ni-1[ClO$_4$]** S24
Figure S19. GC-MS of the CPE experiment with **PhSSPh** and **PA** without **Ni-1[ClO$_4$]** S25
Computational Data: ……………………………………………………………………………….S25
Figure S20. GC-MS of the CPE experiment of **PhSSPh** with **Ni-1[ClO$_4$]** and **cumene** S25
Figure S21. Optimized structures of **Ni-1[ClO$_4$]** S26
Table S4. Cartesian coordinates of the DFT optimized ground-state structure of **Ni-1$^+$**.......S26
Table S5. Cartesian coordinates of the DFT optimized ground-state structure of **Ni-1**. S31
References:...S36

Materials and Methods

General: Chemicals were of reagent grade quality or better, obtained from *Sigma-Aldrich*, *Merck* or *Fluka* and used without further purification. Vitamin B_{12} was a generous gift from *DSM Nutritional Products AG* (Basel/Switzerland), Prof. em. B. Jaun (ETH, Switzerland) and Prof. em. B. Kräutler (University of Innsbruck Austria). All solvents were of reagent, analytical, gradient or UPLC-MS grade, and obtained from commercial suppliers. Bi-distilled H_2O was used in isolation and purification steps. H_2O from a Milli-Q (*Merck-Millipore*) water purification system was used for UPLC-MS measurements and when indicated. Reactions were carried out under N_2 (g) or Ar (g) and monitored for completion by analyzing a small sample (after suitable workup) by TLC or UPLC-MS. Extracts were washed with brine, followed by H_2O and dried over anhydrous Na_2SO_4 or passed through oven-dried cotton. Evaporation of the solvents *in vacuo* was done with the rotary evaporator at given bath temperature and pressure.

UPLC-MS: Samples (2 µL injection) were analysed with a *Vanquish™ Horizon UHPLC* System (*Thermo Fisher Scientific*, Waltham, USA) connected to a *Vanquish™* eλ detector and ISQ-EM ESI mass spectrometer (*Thermo Fisher Scientific*, Waltham, USA), operated in positive or negative mode; scan range *m/z* 200–1500. Separation was performed with an *Acquity BEH C18* HPLC column (1.7 µm particle size, 2x100 mm, *Waters*) kept at 40 °C. The mobile phase consisted of A: H_2O + 0.1% HCOOH and B: CH_3CN + 0.1% HCOOH. UV spectra were recorded between 190 and 670 nm at a 4 nm resolution and at 5 Hz. The mass spectrometer was operated in the positive (negative) electrospray ionization mode at 3000 V (-2’000 V) capillary voltage with a N_2 sheath gas pressure of 41.9 psi, auxiliar gas pressure of 5.5 psi and sweep gas pressure of 0.1 psi. Vaporizer temperature was 238 °C. Spectra were acquired in the mass range from m/z 150 to 2’000 collecting two points s^{-1}, Method: a gradient (0 min 25.0% B, 0.5–2.0 min 25–95% B, 4.01–5.0 min 100%B) using a flow rate of 0.5 mL min^{-1}.

GC-EI-MS: *Trace 1300* GC (*ThermoFisher Scientific*, Milano, Italy) connected to a *Thermo Scientific ISQ* EI-MS (*Walnut Creek, CA, USA*); injection of 1 µL sample (in ether/MeCN 1:1); split injector at 250 °C; He (carrier gas) at 1 mL min^{-1}; *Zebron ZB-5ms* capillary column (*Phenomenex,* USA), 15 m length, 0.25 mm i.d., 0.25 µm film thickness; gradient 20 °C min^{-1} from 60–200 °C, 50 °C min^{-1} from 200–250 °C, then isothermal for 3 min; EI at 70 eV; single stage quadrupole mass analyzer; mass range 50–600 *m*/*z* at 2 scans s^{-1}; data given in *m/z* (rel.%).

High-Resolution ESI-MS: Measurements were performed on a Dionex Ultimate 3000 UHPLC system (*Thermo Fischer Scientific*, Germering, Germany) connected to a QExactive MS with a heated ESI source (*Thermo Fisher Scientific*, Bremen, Germany); onflow injection of 1 µL sample (c = approx. 50 µg mL^{-1} in the indicated solvent) with an XRS auto-sampler (CTC, Zwingen, Switzerland); flow rate 120 µL min^{-1}; ESI: spray voltage 3.0 kV, capillary temperature 280 ˚C, sheath gas 30 L min^{-1}, aux gas 8 L min^{-1}, s-lens RF level 55.0, aux gas temperature 250 ˚C (N_2); full scan MS in the alternating (+)/(-)-ESI mode; mass ranges 80–1200 m/z, 133–2000 m/z, or 200–3000 m/z at 70000 resolution (full width half-maximum); automatic gain control (AGC) target of 3.00 × 106; maximum allowed ion transfer time (IT) 30 ms; mass calibration to <2 ppm accuracy

with Pierce® ESI calibration soln. (Thermo Fisher Scientific, Rockford, USA); lock masses: ubiquitous erucamide (m/z 338.34174, (+)-ESI) and palmitic acid (m/z 255.23295, (-)-ESI).

Chromatography: Column chromatography: Silica gel *60* (40–63 µm, *Merck*) with the indicated solvent system. Thin layer chromatography (TLC): TLC plates (*Merck*); silica gel *60* on aluminum with the indicated solvent system; the spots were visualized by UV light (254 and 366 nm) or were colorful. Preparative TLC: *Merck* TLC Silica gel glass plates (20 × 20 cm). Method: TLC plates were impregnated with $NaClO_4$ (0.1 M), dried at 110 °C for 20 min, mobile phase of CH_2Cl_2/ethylacetate/MeOH (1:4.5:0.2) was used. The bands containing the compound of interest were "scraped" from the TLC plate and the compound was eluted with CH_2Cl_2/CH_3OH (10:1). The solvent of the combined solution was evaporated *in vacuo*, the solid re-dissolved in CH_3CN/H_2O (1:2), then lyophilized.

Solid Phase Extraction (SPE): *Chromafix C18ec* columns from *Macherey-Nagel* were applied for SPE. The compounds were dissolved in CH_3CN/H_2O (1:2) or MeOH/H_2O (1:1), transferred to the adsorbent. The immobilized crude product was washed with H_2O (10 mL), an aq. soln. of $NaClO_4$ (0.2 M, 10 ml), H_2O (10 mL) and subsequently eluted with CH_3CN (5 mL) from the solid-support.

Spectroscopy: UV/Vis spectra: *Cary Series* spectrophotometer (*Agilent* technologies) or *Specord 250 Plus* (*Analytik Jena*) using 1 cm quartz cuvettes (*Hellma Analytics*); samples in CH_2Cl_2 or CH_3CN; λ_{max} (log ε) in nm. NMR spectroscopy: ^1H- and ^{13}C-NMR as well as 2D-NMR spectra were recorded on a 500 MHz *Bruker Avance 500 NMR* equipped with a *BOSS-II* shim system, a digital lock control unit, an *AMOS* Control System, a *DQD* unit, a *BVT3000* with a *BCU05* cooling unit and *GRASP Level II* for gradient spectroscopy, using a 5 mm BB inverse probe head (^1H) with an actively shielded z-gradient coil and ATM or a CRYO 5 mm QNP probe head (^1H,^{13}C) with an actively shielded z-gradient coil and ATM. Spectra were recorded at 300 K, TMS was used as a reference for ^{13}C- and 2D-NMR experiments. Chemical shifts in ppm rel. to $CDCl_3$ (δ_{CHCl3} = 7.26 ppm). All the NMR data were processed with *MestReNova*.

Electrochemistry: Cyclic voltammetry was performed in dry CH_3CN (analytical grade; kept over 3 Å molecular sieve) and $TBAPF_6$ (both *Acros Organics*). Working electrode: 2 mm glassy carbon disk; counter electrode: Pt rod; reference electrode: Ag/AgCl non aqueous; *797 VA* Computrace (all *Metrohm Schweiz AG*). The potential of the reference electrode was calibrated against the ferrocene/ferrocenium (Fc/Fc^+) couple before and after each experiment. The working electrode was polished with 0.3 µm alumina powder/MeOH paste before each experiment. Scan rates, and concentration of **Ni-1[ClO$_4$]** as indicated. Electrolyte soln. of $TBAPF_6$ (0.1 M) in CH_3CN was purged with N_2(g) for 20 min. A blank scan was recorded at each scan rate before addition of **Ni-1[ClO$_4$]**. The soln. was purged for 15 min before three cyclic were scanned in the indicated potential range. Electrocatalysis: It was probed with cyclic voltammetry of **Ni-1[ClO$_4$]** (6.012 mg, 1 mM) in electrolyte soln. (5.0 mL) of $TBAPF_6$ (0.1 M) in CH_3CN. Titrations with increasing concentration of diphenyl disulfide (**PhSSPh**) (1–110 mM; 1–110 equiv.) were conducted by addition of the respective amount of substrate into the bulk soln.. It was purged with N_2 (15 min) before recording each scan. CPE: Working electrode: 2 mm glassy carbon rod; counter electrode: Pt mesh (all *Metrohm Schweiz AG*); reference electrode: Ag/AgCl non-aqueous electrode, 59 × 4.5 mm (*ALS Japan*). The potential of the reference electrode was calibrated against the ferrocene/ferrocenium (Fc/Fc^+) redox couple before and after each experiment; *BioLogic*

Computrace. Electrolyte soln. of $TBAPF_6$ (0.1 M) in MeCN/water (16% v/v) was used in all the performed experiments (1 mL in each compartment of the H-cell). Working electrode, reference electrode, **Ni-1**[ClO_4] (1 mM), and **PhSSPh** or **PA** as indicated for each experiment were added in one compartment of the H-cell. The counter electrode in the other compartment was separated by a P4 fritted glass. Compartments were purged with N_2 (g) before applying any potential. Applied potential in all the electrocatalysis experiments is -1.65 vs. Fc/Fc^+. Both compartments were stirred and kept under inert atmosphere. The reaction time was 90 min if not stated otherwise. After the experiment, an aliquot (0.5 mL) of the reaction mixture was diluted with H_2O (0.5 mL) and washed with Et_2O (3 x 2 mL). Samples of the combined organic phases were further diluted for GC-MS analysis as described under *Quantitative GC-Measurements/ Quantification.*

Quantitative GC-Measurements: Calibration curve: A soln. of the internal standard (**IS**) 1,3,5-trimethoxy benzol (1,3,5-TMB, R_t= 8.24 min; m/z = 168.1; 9.98 mg L^{-1}, 0.05933 mmol L^{-1}; soln. *A*) in Et_2O was prepared. Soln. A was subsequently used for the preparation of the stock soln. of thiophenol (**PhSH**, R_t= 3.77 min; m/z =110.1; 16.4 mg L^{-1}, 0.147 mmol L^{-1}; soln. *B*), and for any further dilution of soln. B. For recording the calibration curve, five samples with [**PhSH**] = 0.1473, 0.0736, 0.0368, 0.0184, 0.0036, 0.0018 mmol L^{-1} and [**IS**] = 0.05933 mmol L^{-1} were prepared. GC-MS spectra of each sample (1 mL) were recorded and the area of the peaks of **PhSH** (R_t= 3.77 min) and **IS** (R_t= 8.24 min) were determined with the *Chromeleon 7.3.2* software. Ratios of $Area_{PhSH}/Area_{IS}$ were plotted against [**PhSH**]/[**IS**] yielding equation 1.

$$[\mathbf{PhSH}]/[\mathbf{IS}] = 1.6646\ (Area_{PhSH}/Area_{IS}) + 0.0789 \qquad (1)$$

Quantification*:* An aliquot (0.5 mL) of the combined organic phases after workup was diluted with MeCN (3.5 mL). A GC-MS spectrum of an aliquot (1 mL) of this soln. was measured and the area of the signal of **PhSH** (R_t= 3.77 min; m/z =110.1) was determined. To quantify $[\mathbf{PhSH}]_{Sample}$ within the reaction mixture of an CPE experiment, equation 2 is used. It is derived from equation 1 considering the dilution factor of 16 of the sample during work-up and sample preparation.

$$[\mathbf{PhSH}]_{Sample} = ([\mathbf{IS}] \times (1.6646\ (Area_{PhSH}/Area_{IS}) + 0.0789) \times 16 \qquad (2)$$

Spectroelectrochemistry: A soln. of **Ni-1[ClO_4]** (1.5 mg, 2.56 mM) in CH_3CN (0.5 mL, containing 0.6 M $TBAPF_6$) was transferred into an optically transparent thin layer electrochemical (OTTLE) cell with a Pt-mesh working and counter electrode and an Ag quasi-reference electrode. The applied potential was calibrated externally using the Fc/Fc^+ redox couple in $TBAPF_6$ soln. 0.6 M in CH_3CN. All potentials given in V *vs.* Fc/Fc^+. The cell was equipped with CaF_2 windows. UV/Vis spectra (200–1000 nm) were recorded using a *Cary Series* spectrophotometer (*Agilent* technologies). Potentials were applied by a *Vertex* potentiostat (*Ivium*). A trigger scheme was used to synchronize the electrochemical data acquisition (*IviumSoft*).

EPR Experiment: Sample Preparation: Reductions were carried out in a mixture of toluene/acetonitrile (2:3 mL) containing $TBAPF_6$ (0.1 M) , in a two-compartment electrolysis cell under an Ar(g) atmosphere. Compartments were separated by *DURAPURE®* membrane filters (filter type *0.2 µm GVHP*). The cathode compartment contained **Ni-1[ClO_4]** (13.1 mg, 2.24 mM) in electrolyte soln. (5 mL), a platinum mesh as working electrode, and a silver wire as a pseudo-reference electrode. The anode compartment contained a coiled silver wire as counter electrode and electrolyte solution. Prior to electrolysis, a cyclic voltammetry scan was performed to identify the peak potential of the first reduction wave of **Ni-1[ClO_4]**. For electrolysis, the working potential was set slightly more negative (ca. 50 mV) than this peak potential, in order to avoid over-

reduction. During potentiostatic electrolysis, the current was monitored. The initial current dropped and asymptotically approached a limiting value after 90 min, which was interpreted as completion of the desired reduction. Reduction was accompanied by a change of color from orange to orange-brown in the cathode compartment solution. The soln. was then collected with a gas tight syringe conditioned Ar(g) and it was transferred into a quartz EPR tube. The sample was immediately frozen in N_2 (l) and kept there until it was mounted in the spectrometer. EPR tube was sealed under *vacuum* connected to a Schlenk line. Data Collection: EPR data collected by continuous-wave electron paramagnetic resonance (CW-EPR) spectroscopy experiments at 10 K on a Bruker Elexsys E500 spectrometer operating at X-band frequencies, using an ER4102ST microwave resonator and equipped with an Oxford helium (ESR900) cryostat. All CW-EPR spectra were acquired at room temperature with the following spectrometer parameters: microwave frequency = 9.4 GHz, sweep width = 590 mT, center field = 300 mT, modulation frequency = 100 kHz, modulation amplitude = 3 G, microwave power = 2.007 mW, power attenuation = 20 dB, conversion time = 163.84 ms, time constant = 40.96 ms. All measured g-factors were offset-corrected against a known standard (i.e., free radical 1,1-diphenyl-2-picrylhydrazyl). The spectra were simulated by using the Easyspin[S5] package running on Matlab.

Computational Details: Density functional theory (DFT) calculations were carried out using the *Gaussian 09* program package.[S6] The TPSS density functional[S7] was used in association with the Stuttgart/Dresden effective core potentials (SDD) basis set[S8] for the Ni atom, with the polarized single-zeta def2-SVP basis set[S9,S10] for the remaining atoms, and with the conductive polarizable continuum model (CPCM)[S11,S12] for the solvent effects (water). The optimized ground state structures were obtained by restricted or unrestricted calculations and confirmed to be potential energy minima by vibrational frequency calculations at the same level of theory.

Reaction Schemes

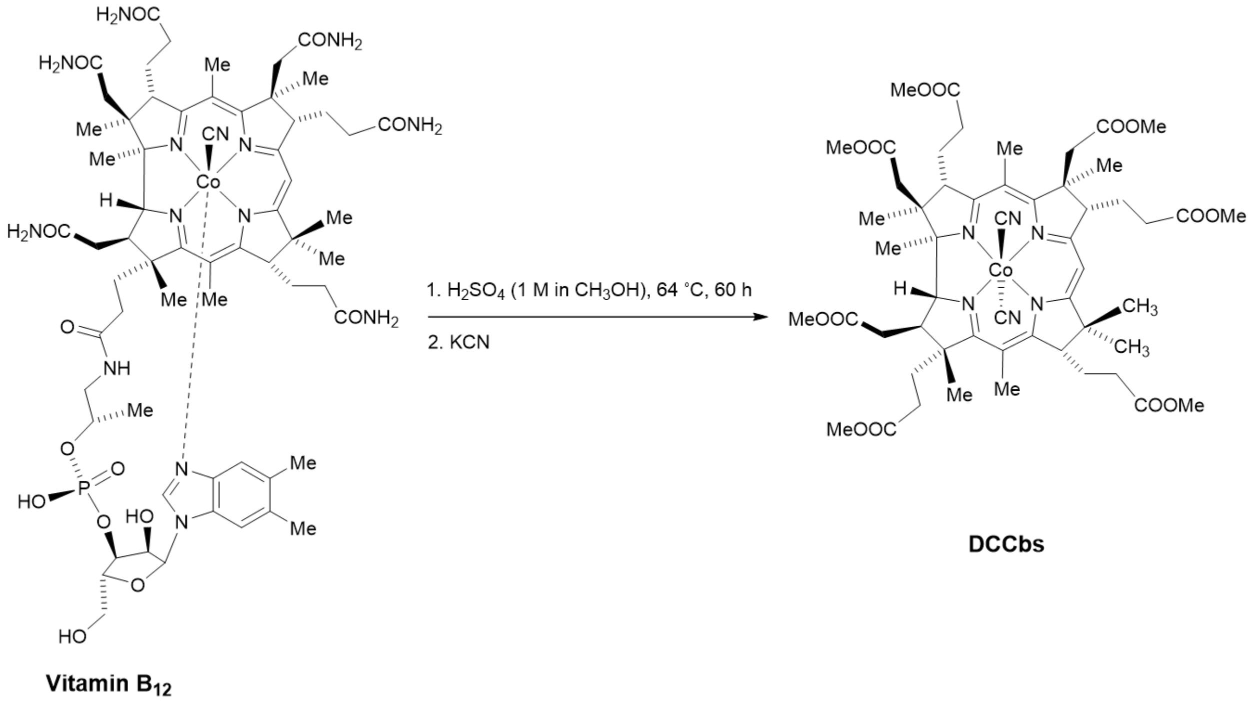

Scheme S1. Synthesis of **DCCbs**.[S1,S2]

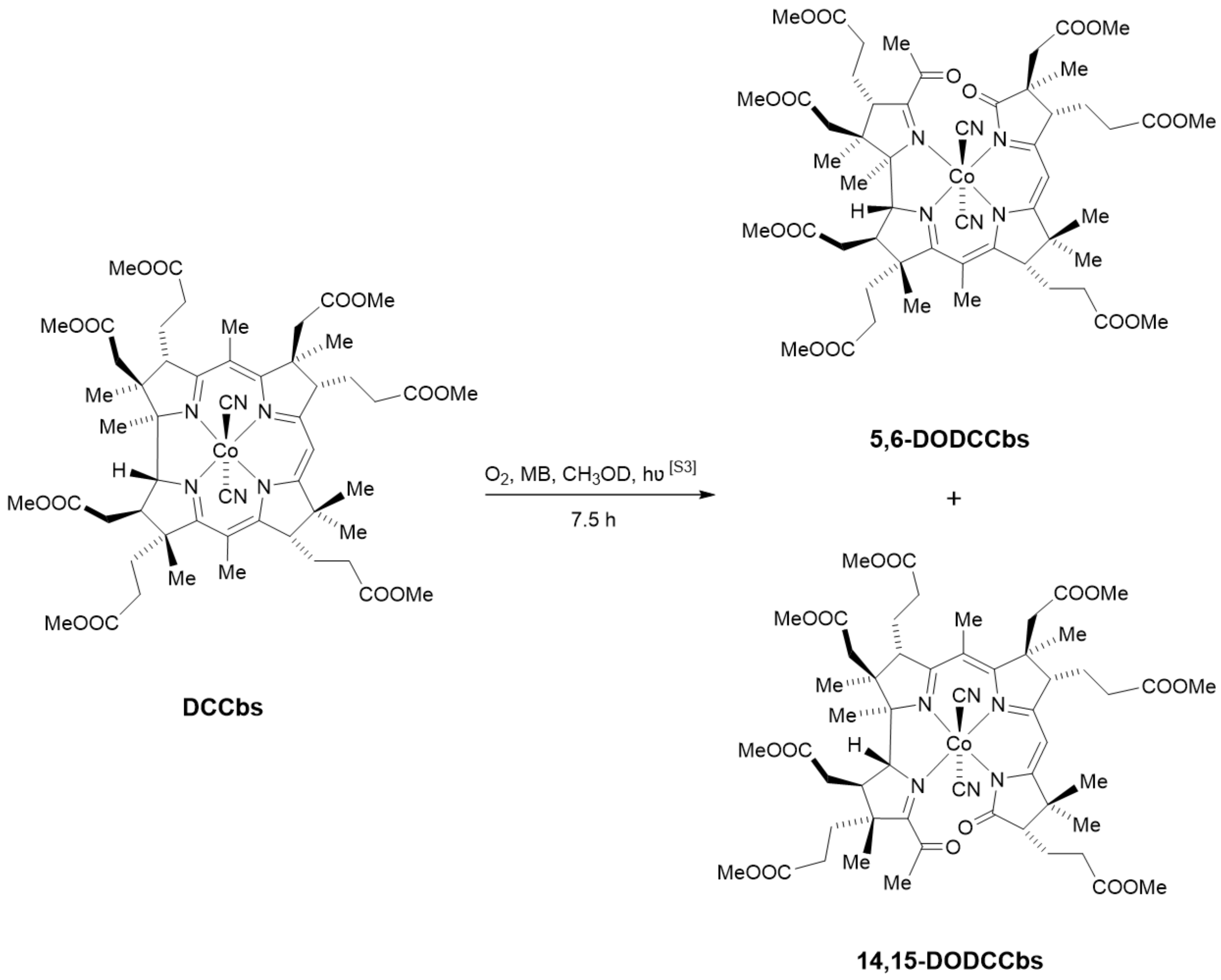

Scheme S2. Photo-oxygenolysis of **DCCbs** to generate **5,6-DODCCbs**.[S3]

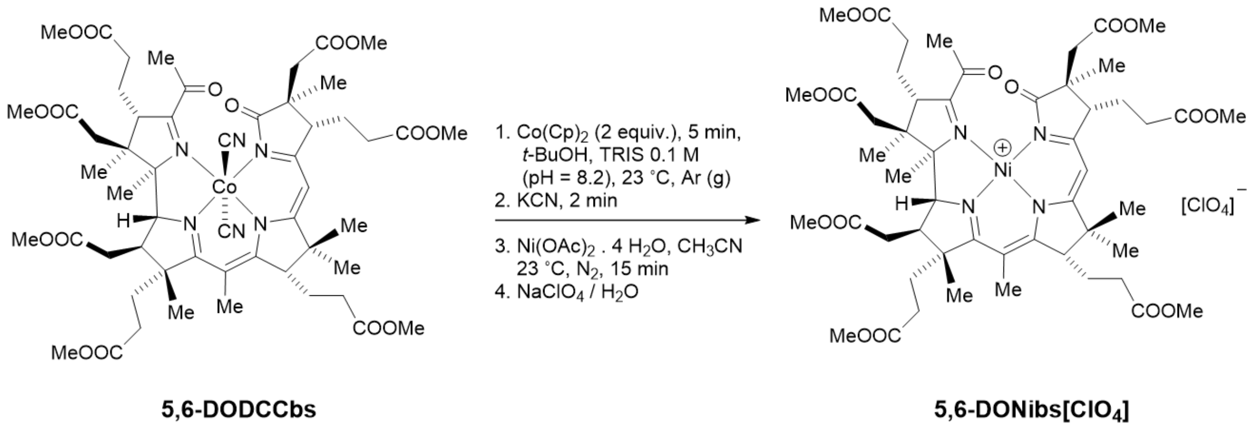

Scheme S3. Synthesis of **5,6-DONibs[ClO$_4$]** from **5,6-DODCCbs**.[S4]

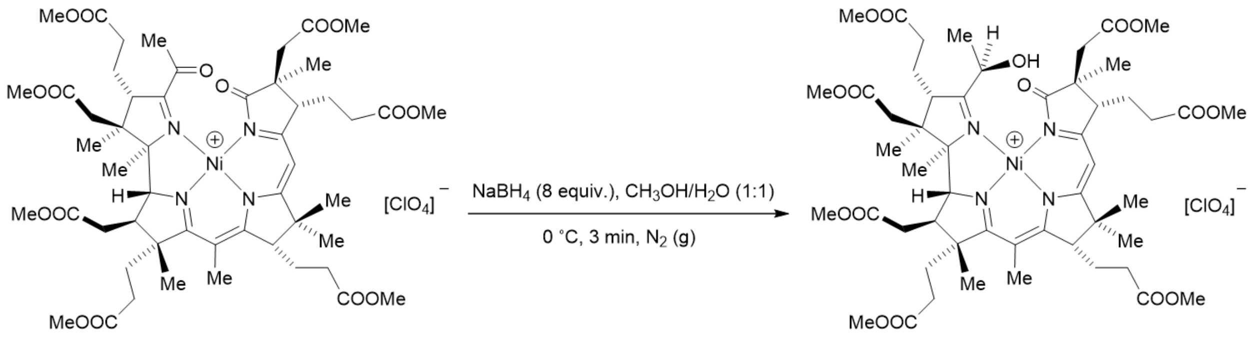

5,6-DONibs[ClO$_4$] **Ni-1[ClO$_4$]**

Scheme S4. Synthesis of **Ni-1[ClO$_4$]** from **5,6-DONibs[ClO$_4$]**.

Experimental Procedure:

Co$_{\alpha\beta}$-dicyano heptamethyl cobyrinate (DCCbs):

DCCbs was synthesized and isolated as described in S4 following a route of Werthemann[S1] with modifications described by Marques *et al.*[S2] *Co$_{\alpha\beta}$-dicyano heptamethyl cobyrinate*: **^{1}H-NMR** (CD_3OD, *c* = 5.4 · 10^{-3} M): 5.81 (*s*, *H*C10), 3.86 (*d*, *J* = 10.5, *H*C19), 3.84–3.82 (*m*, *H*C3), 3.81 (*s*, *H_3*CO_2C182), 3.76 (*s*, *H_3*CO_2C22), 3.75 (*s*, *H_3*CO_2C72), 3.73 (2 *s*, *H_3*CO_2C33, *H_3*CO_2C173), 3.71 (*s*, *H_3*CO_2C83), 3.65 (*s*, *H_3*CO_2C133), 3.47 (*dd*, *J* = 8.0, 4.9, *H*C8), 3.24 (*dd*, *J* = 6.0, 4.4, *H*C13), 2.90 (*dd*, *J* = 10.6, 3.6, *H*C18), 2.85–2.75 (*m*, *H_a* of H_2C71, *H_2*C181), 2.74–2.57 (*m*, *H_a* of H_2C21, H_2C172, *H_2*C32), 2.57–2.38 (*m*, *H_b* of H_2C71, H_2C21, *H_a* of H_2C171, *H_2*C82), 2.34 (*s*, *H_3*C151), 2.32– 2.21 (*m*, *H_b* of H_2C172, H_2C132, *H_a* of H_2C81, H_2C131, *H_2*C31) superimposed by 2.29 (*s*, *H_3*C51), 1.91–1.82 (*m*, *H_b* of H_2C131, H_2C171), 1.74 (*dt*, *J* = 8.5, 4.5, *H_b* of H_2C81), 1.66 (*s*, *H_3*C7A), 1.53 (*s*, *H_3*C1A), 1.46–1.45 (2 *s*, *H_3*C2A, *H_3*C12A), 1.32 (*s*, *H_3*C17B); 1.32 (*s*, *H_3*C12B) **UPLC-MS** (MS^+): R_t = 2.22 min and 2.32 min, *m/z* =1062.53 (100, [*M* - CN]$^+$, *m/z$_{calc}$*: 1062.45 for $C_{53}H_{73}CoN_5O_{14}^+$).

5,6-dioxo-5,6-*seco* Co$_{\alpha\beta}$-dicyano heptamethyl cobyrinate (5,6-DODCCbs):

Photo-oxidation of **DCCbs** (179 mg, 164 μmol) was performed as described in S4 based on a procedure developed by Kräutler and Stepánek.[3] Products were isolated as **5,6-DODCCbs** (75.0 mg, 66.9 μmol, 41%) and 14,15-dioxo *Co$_{\alpha\beta}$-dicyano heptamethyl cobyrinate* (**14,15-DODCCbs**; 60.7 mg, 54.1 μmol, 33%). *5,6-dioxo-5,6-seco Co$_{\alpha\beta}$-dicyano heptamethyl cobyrinate*: **^{1}H-NMR** ($CDCl_3$, *c* = 4.5 · 10^{-3} M): 5.55 (*s*, *H*C10), 3.81–3.65 (*m*, *H*C19, *H*C3) superimposed by 3.78 (*s*, O*CH_3*), 3.74 (*s*, O*CH_3*), 3.72 (*s*, O*CH_3*), 3.69 (2 *s*, 2 O*CH_3*) and 3.67 (*s*, O*CH_3*), 3.58 (*s*, O*CH_3*), 3.27 (*dd*, *J* = 8.6, 3.2, 1 H, corrin-C*H*), 3.00 (*app. d*, *J* = 4.2, 1 H, corrin- C*H*), 2.91 (*t*, *J* = 4.9, corrin-C*H*), 2.77–2.28 (*m*, 12 corrin-C*H* and corrin-C*H_2*) superimposed by 2.66 (*s*, *H_3*C51), 2.25–2.18 (*m*, 7 corrin-C*H* and corrin-C*H_2*) superimposed by 2.21 (*s*, *H_3*C151) and 1.99 (*s*, corrin-C*H_3*), 1.76–1.63 (*m*, 4 corrin-C*H_2*), 1.30 (*s*, corrin-C*H_3*), 1.26 (*s*, corrin-C*H_3*), 1.24 (*s*, corrin-C*H_3*), 1.20 (*s*, corrin-C*H_3*), 1.00 (*s*, corrin-C*H_3*) **UPLC-MS** (MS^+) : R_t = 2.20 min, *m/z* =1094.52 (100, [*M* – CN]$^+$, *m/z$_{calc}$*: 1094.44 for $C_{53}H_{73}CoN_5O_{16}^+$).

5,6-dioxo-5,6-*seco*-heptamethyl nibyrinate perchlorate (5,6-DONibs[ClO_4]):

5,6-DONibs[ClO_4] was synthesized and isolated as described in S4. *5,6-dioxo-5,6-seco-heptamethyl nibyrinate perchlorate*: **UV/Vis** (CH_3CN, c = 2.5 · 10^{-5} M): 259 (3.88), 276 (3.93), 318 (*sh.*, 3.71), 358 (*sh.*, 3.54), 459 (3.79). **^{1}H-NMR** ($CDCl_3$, c = 4.6 · 10^{-3} M): 5.74 (*s*, *H*C10), 3.93 (*d*, *J* = 11.0, *H*C19), 3.75 (s, OC*H*$_3$), 3.73 (*s*, OC*H*$_3$), 3.73–3.65 (*m*, corrin-C*H*) superimposed by 3.70, 3.71 (2 *s*, 2 OC*H*$_3$), 3.68 (*s*, OC*H*$_3$), 3.66 (*s*, OC*H*$_3$) and 3.64 (*s*, OC*H*$_3$), 3.04–3.01 (*m*, corrin-C*H*), 2.99 (*s*, *H*$_3$C51), 2.95 (*t*, *J* = 5.1, corrin-C*H*), 2.89–2.84 (*m*, corrin- C*H*), 2.77–2.70 (*m*, 2 corrin-C*H*), 2.62 (*d*, *J* = 14.7, corrin-C*H*), 2.59–2.45 (*m*, corrin-C*H*, 2 corrin-C*H*$_2$), 2.44–2.39 (*m*, corrin-C*H*$_2$), 2.20 (*s*, *H*$_3$C151), 2.11 (*s*, corrin-C*H*$_3$) , 2.09–2.03 (*m*, corrin-C*H*$_2$), 2.00–1.85 (*m*, 3 corrin-C*H*$_2$), 1.75–1.66 (*m*, 2 corrin-C*H*$_2$), 1.39 (*s*, corrin-C*H*$_3$), 1.32, 1.31 (2 superimposed *s*, 2 corrin-C*H*$_3$), 1.19 (*s*, corrin-C*H*$_3$), 0.97 (*s*, corrin-C*H*$_3$). **UPLC-MS** (MS^+): R_t = 2.49 min, *m/z* =1067.54 (100, M^+, m/z_{calc}: 1067.44 for $C_{52}H_{73}N_4NiO_{16}^+$).

(5R)-5-hydroxy-6-oxo-5,6-*seco*-heptamethyl nibyrinate perchlorate (Ni-1[ClO_4]):

5,6-DONibs[ClO_4] (18.5 mg, 15.9 µmol, 1 equiv.) was dissolved in a mixture of H_2O/MeOH (1:1, 18 mL) and purged with N_2 (g) for 10 min. Upon the addition of $NaBH_4$ (2.55 mg, 67.4 µmol, 4 equiv.) under an N_2 (g) atmosphere in an ice-water bath, the color of the mixture turned immediately from orange to dark orange. Stirring at 0°C was continued until reaction control by UPLC-MS indicated full conversion (3 min). Excess of $NaBH_4$ was quenched with an aq. soln. of CH_3COOH (0.2 M; *ca*. 2 mL) until no more formation of H_2 (g) was observed. The reaction mixture was subsequently purified by SPE. The organic solvent was removed under reduced pressure at 100 mbar and 20 ˚C. The crude product was purified with prep. TLC to afford a highly pure **Ni-1[ClO_4]** (16.3 mg, 88% yield). *(5R)-5-hydroxy-6-oxo-5,6-seco-heptamethyl nibyrinate perchlorate*: **UV/Vis** (CH_3CN, c = 1.12 · 10^{-5} M): 215 (4.43), 276 (4.14), 320 (3.38), 356 (3.55), 461(3.94). **^{1}H-NMR** (500 MHz, $CDCl_3$, c = 9.6 · 10^{-3} M, 300 K): 5.73 (*s*, *H*C10), 4.86 (*q*, *J* = 6.52, *H*C5), 3.75 (*s*, OC*H*$_3$), 3.71 (*s*, OC*H*$_3$), 3.7 (*s*, OC*H*$_3$), 3.69 (*s*, OC*H*$_3$), 3.67 (*s*, OC*H*$_3$), 3.66 (2*s*, OCH_3), 3.57 (*d*, *J* = 11.17, *H*C19), 3.54 (*t*, *J* = 5.01, *H*C3), 3.32 (*t*, *J* = 6.47, *H*C8), 2.95 (*t*, *J* = 5.57, *H*C13), 2.87-2.84 (*d*, *J* = 17.28, H_b of *H*$_2$C71), 2.83–2.79 (*m*, *H*C18), 2.60–2.55 (*m*, H_b of *H*$_2$C172, *H*$_2$C82) superimposed by 2.61–2.58 (*d*, *J* = 17.08, H_a of *H*$_2$C71), 2.50–2.42 (m, *H*$_2$C181, *H*$_2$C32), 2.39–2.3 (*m*, H_b of *H*$_2$C21, H_b of *H*$_2$C171), 2.18 (*s*, *H*$_3$C151), 2.14 (*s*, *H*$_3$C1A), 2.08–2.01 (*m*, H_b of *H*$_2$C81, H_b of *H*$_2$C132, H_a of *H*$_2$C21), 2.00–1.93 (*m*, H_a of *H*$_2$C81, H_b of *H*$_2$C131, *H*$_2$C31, H_a of *H*$_2$C132), 1.8 (*d*, *J* = 6.6, C51), 1.68–1.56 (*m*, H_a of *H*$_2$C131, H_a of *H*$_2$C171), 1.42 (*s*, *H*$_3$C12A), 1.34 (*s*, C12B), 1.28 (s, *H*$_3$C17B), 1.21 (*s*, *H*$_3$C2A), 0.93 (*s*, *H*$_3$C7A). **^{13}C-NMR** ($CDCl_3$, 300 K): 194.39 (C4), 189.27 (C6), 180.87 (C11), 175.53 (C9), 173.95, 173.00, 172.99, 172.89, 172.86, 172.23, 171.52 (C=O ester groups), 172.99 (C22), 161.28 (C14), 107.72 (C15), 93.47 (C10), 85.32 (C1), 74.24 (C19), 67.88 (C5), 56.8 (C17), 56.27 (C3), 52.7 (C13), 52.54, 52.39 52.12, 52.11, 52.03, 51.93, 51.89 (CH_3-O ester), 48.46 (C12), 48.33 (C8), 47.82 (C7), 45.11 (C2) 42.11 (C21), 40.82 (C18), 39.23 (C71), 33.44 (C32), 32.68 (C82), 31.5 (C171), 31.08 (C181), 30.71 (C132), 30.56 (C12A), 29.61 (C172), 24.99 (C131), 23.91 (C31), 22.99 (C51), 22.32 (C81), 21.21 (C7A), 20.7 (C12B), 19.88 (C1A), 17.29 (C17B), 16.93 (C2A), 15.22 (C151). **UPLC-MS**: R_t = 2.48 min, *m/z* = 1069.49 (100, M^+, m/z_{calc}: 1069.5 for $C_{52}H_{75}N_4NiO_{16}^+$). **(+)-HR-ESI-MS**: 1069.49084 (100, M^+, m/z_{calc}: 1069.45127 for $C_{52}H_{75}N_4NiO_{16}^+$).

Atom Numbering of Ni-1[ClO_4]:

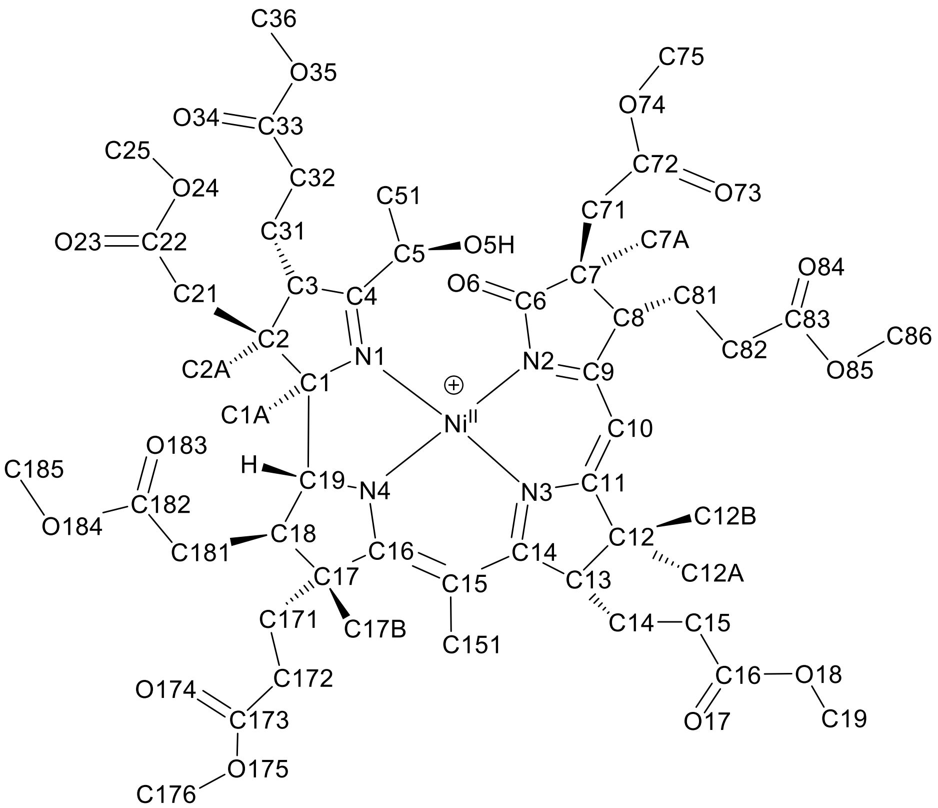

Figure S1. Atom Numbering of **Ni-1[ClO_4]**. Counter ion is eliminated for more clarity.

Analytical Data:

(5R)-5-hydroxy-6-oxo-5,6-*seco*-heptamethyl nibyrinate perchlorate (Ni-1[ClO$_4$]):

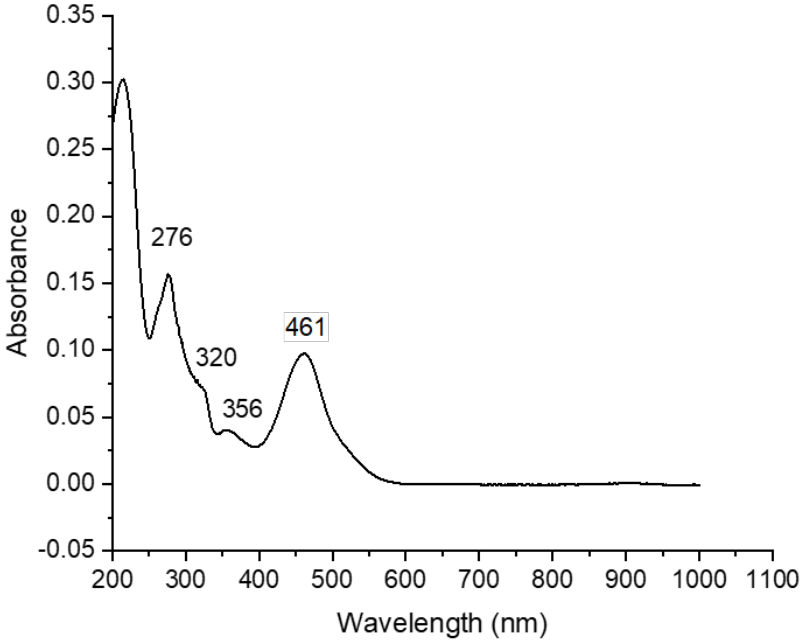

Figure S2. UV/Vis spectrum of **Ni-1[ClO$_4$]** (CH_3CN, 11.2 μM).

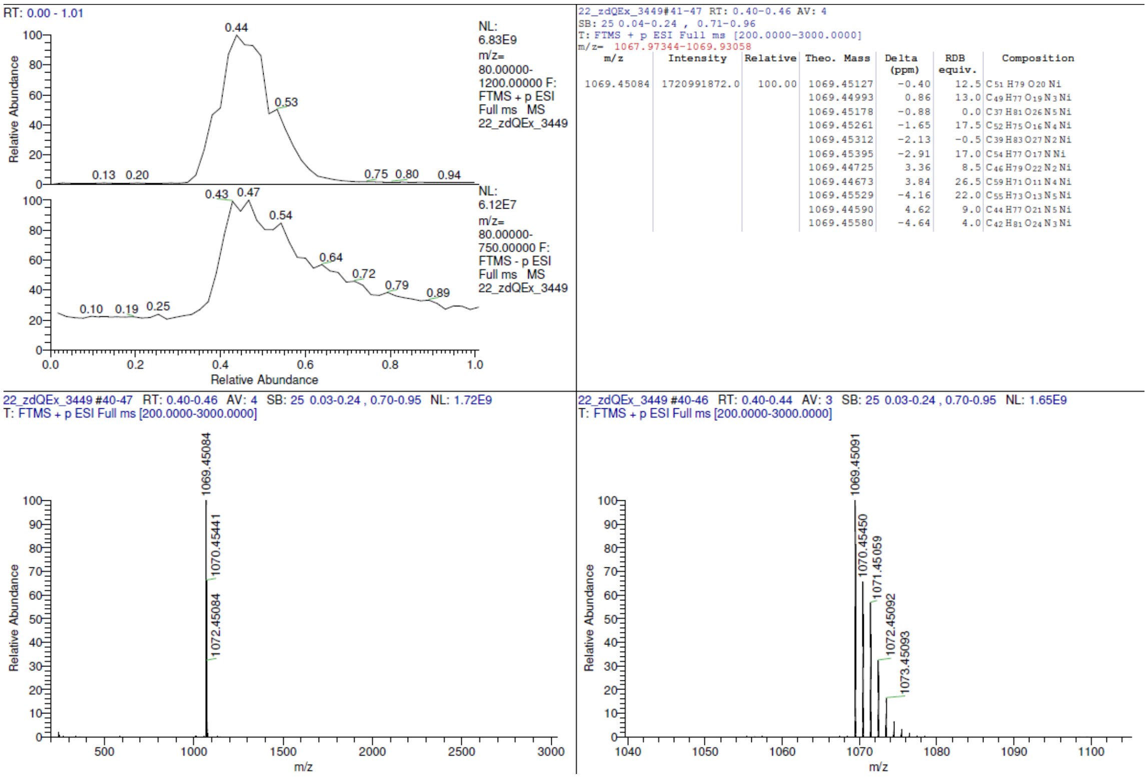

Figure S3. (+)-HR-ESI-MS of **Ni-1[ClO$_4$]**.

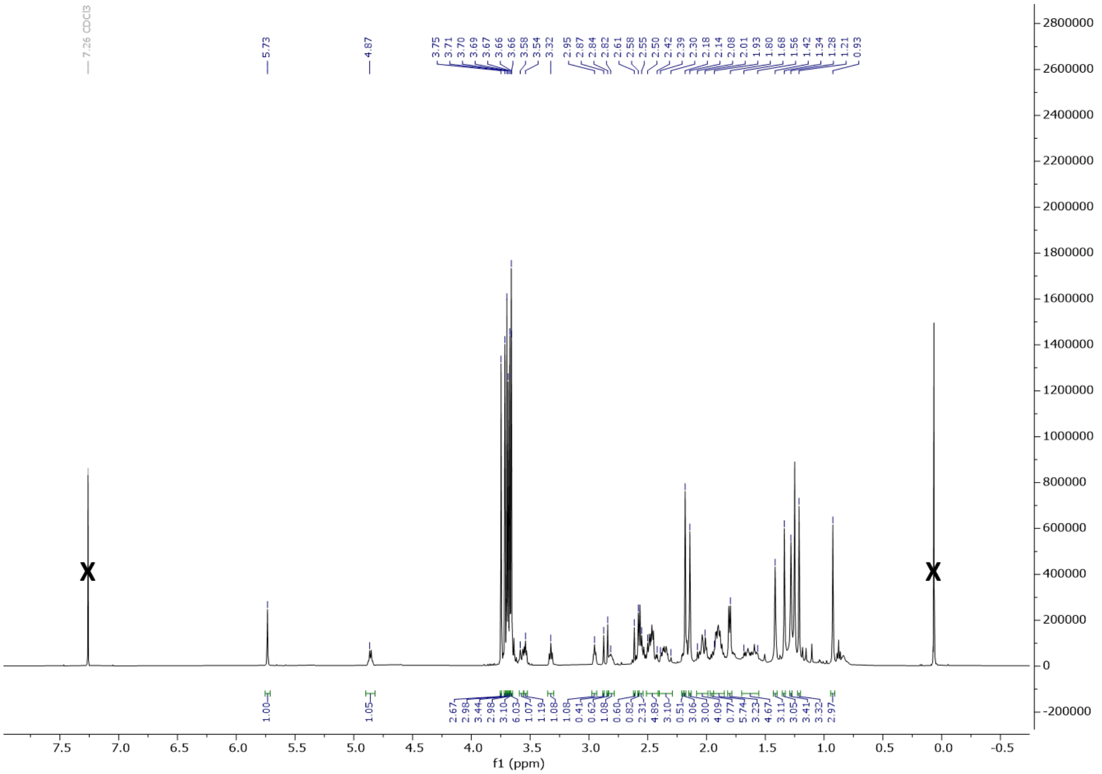

Figure S4. ^{1}H-NMR of **Ni-1[ClO$_4$]**.

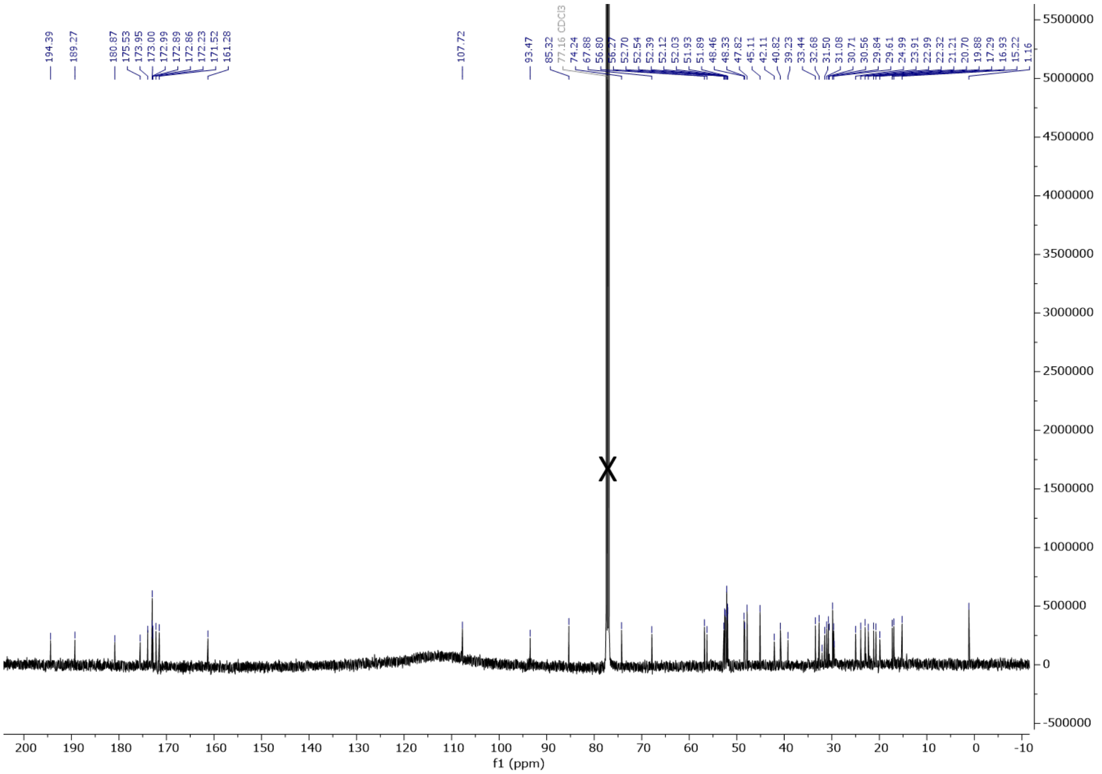

Figure S5. ^{13}C-NMR of **Ni-1[ClO$_4$]**.

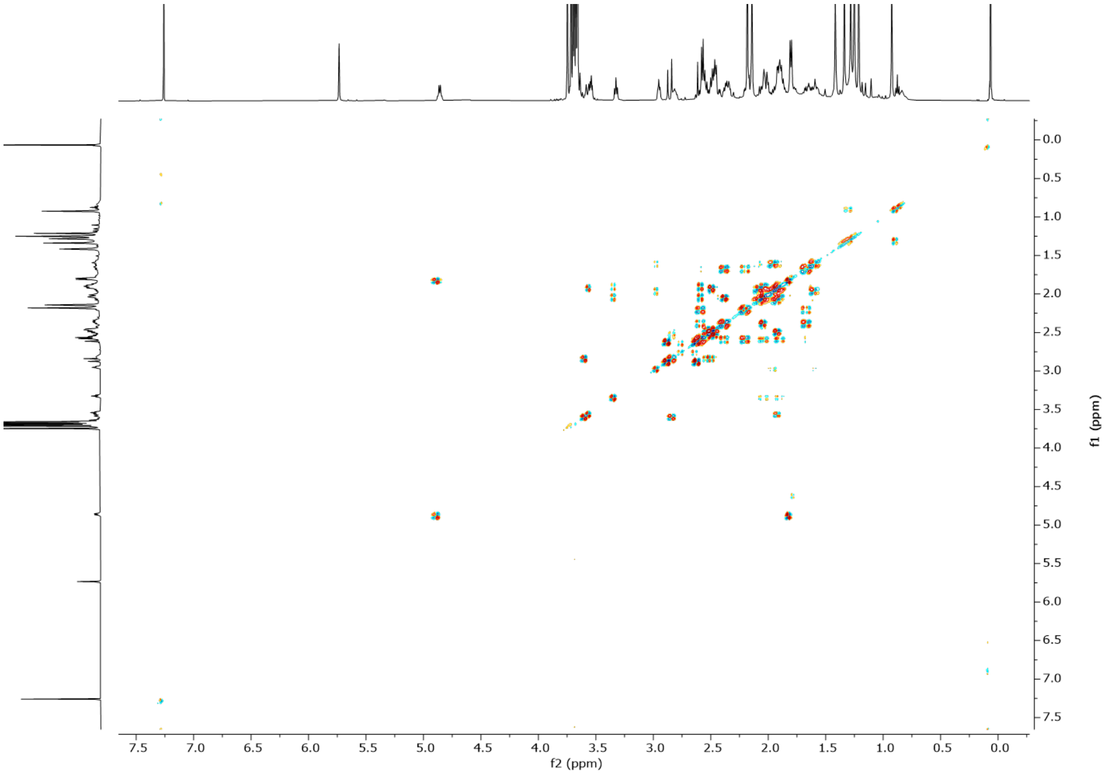

Figure S6. COSY of **Ni-1[ClO$_4$]**.

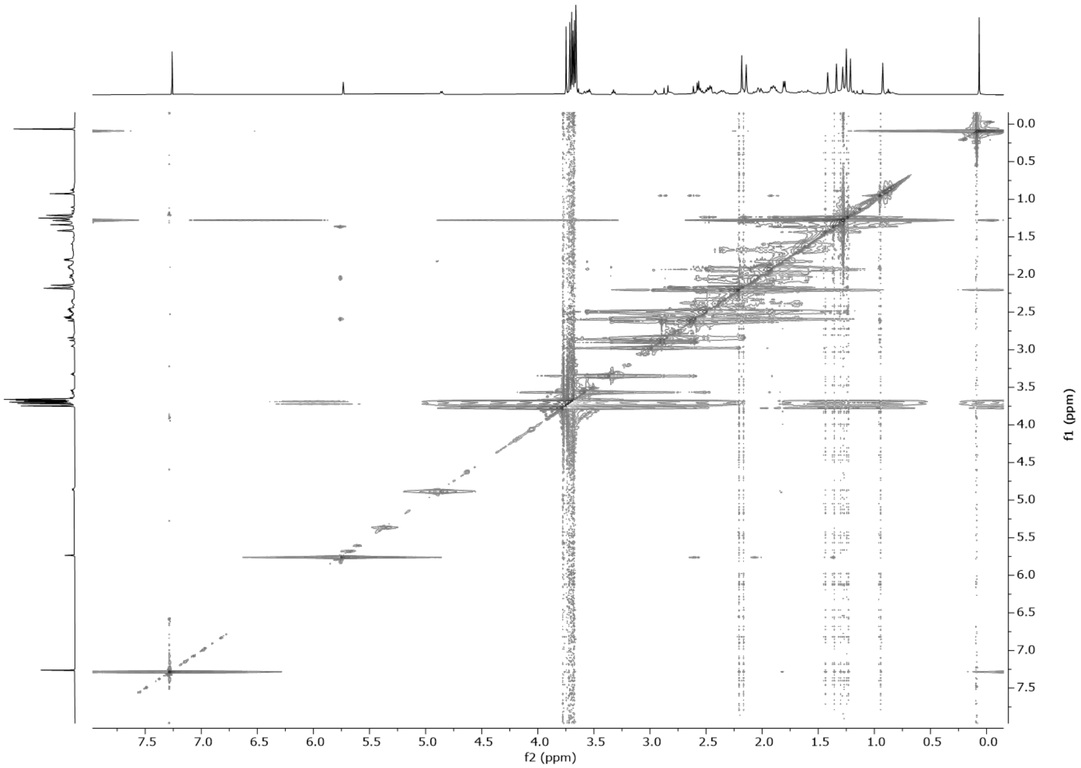

Figure S7. NOESY of **Ni-1[ClO$_4$]**.

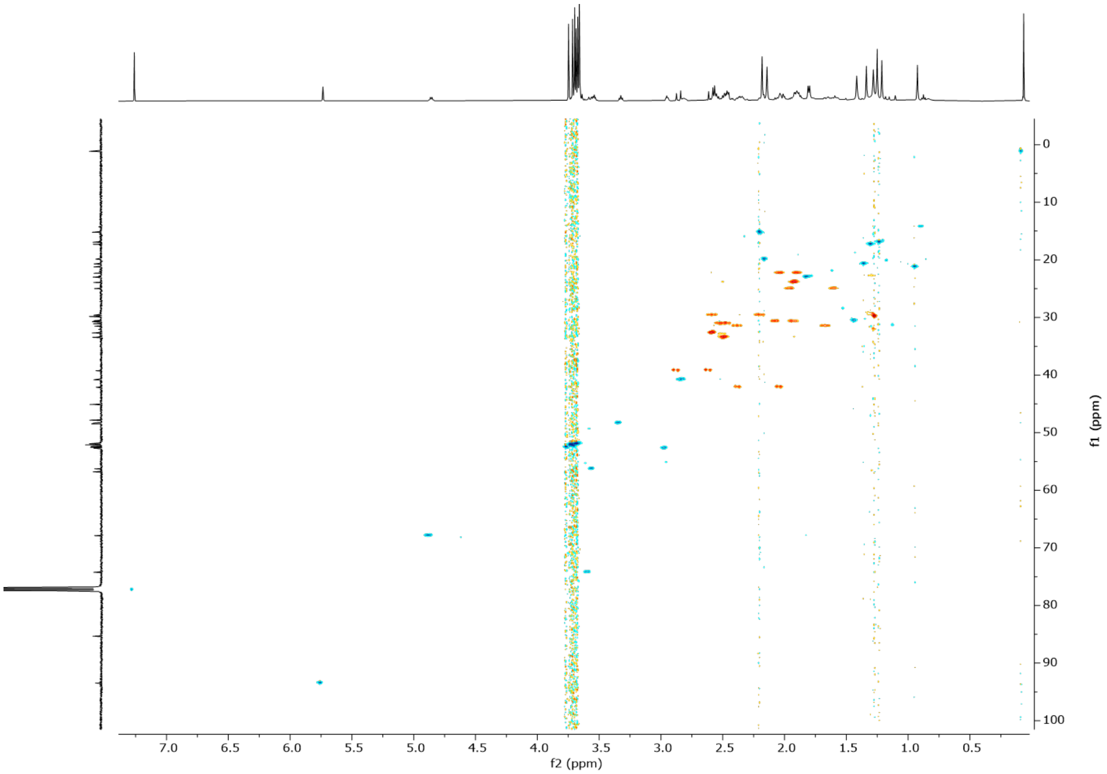

Figure S8. HSQC of **Ni-1[ClO4]**.

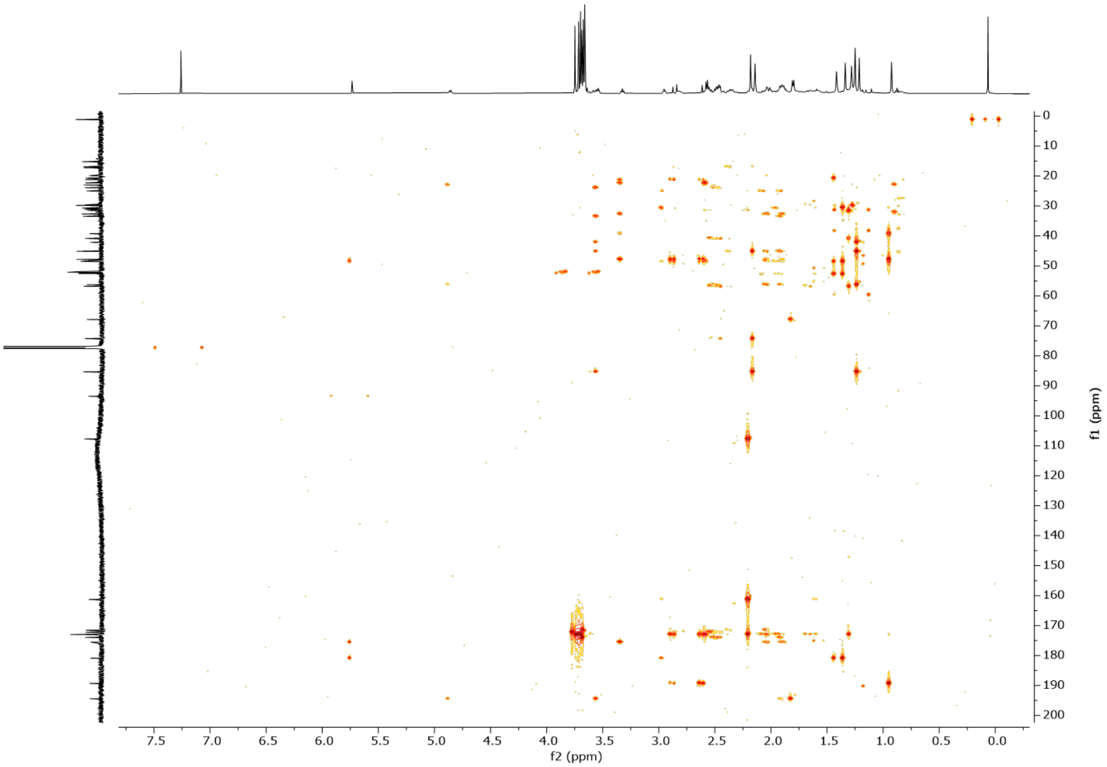

Figure S9. HMBC of **Ni-1[ClO$_4$]**.

Table S1. ^{1}H-NMR chemical shifts and ^{13}C-NMR chemical shifts of **Ni-1[ClO$_4$]**.

Atom	δ ^{13}C [ppm]	δ ^{1}H [ppm]	multiplicity	Coupling constant *J*
**C1**	85.32	-		
**C10**	93.47	5.73	s	
**C11**	180.87	-		
**C12**	48.46	-		
**C12A**	30.56	1.42	s	
**C12B**	20.7	1.34	s	
**C13**	52.7	2.95	t	$J(^{1}H,^{1}H)$ = 5.57 Hz
**C131**	24.99	1.56 1.95	m	
**C132**	30.71	1.9 2.07	m	
**C133**	172.86	-		
**C14**	161.28	-		
**C15**	107.72	-		
**C151**	15.22	2.18	s	
**C16**	172.98	-		
**C17**	56.8	-		
**C171**	31.5	2.35 1.67	m	
**C172**	29.61	2.17 2.46	m	
**C173**	172.99	-		
**C17B**	17.29	1.28	s	
**C18**	40.82	2.82	m	
**C181**	31.08	2.5 2.42	m	
**C182**	172.23	-		
**C19**	74.24	3.57	d	$J(^{1}H,^{1}H)$ = 11.17 Hz
**C1A**	19.88	2.14	s	
**C2**	45.11	-		
**C21**	42.11	2.36 2.05	AB system	$J_a(^{1}H,^{1}H)$ = 13.17 Hz<br>$J_b(^{1}H,^{1}H)$ = 10.31 Hz
**C22**	173.00	-		
**C2A**	16.93	1.21	s	
**C3**	56.27	2.54	t	$J(^{1}H,^{1}H)$ = 5.01 Hz
**C31**	23.91	1.86-1.93	m	
**C32**	33.44	2.45-2.5	m	
**C33**	173.95	-		
**C4**	194.39	-		
**C5**	67.88	4.86	q	$J(^{1}H,^{1}H)$ = 6.5 Hz
**C51**	22.99	1.8	d	$J(^{1}H,^{1}H)$ = 6.6 Hz
**C6**	189.27	-		
**C7**	47.82	-		
**C71**	39.23	2.6 2.86	AB system	$J_a(^{1}H,^{1}H)$ = 16.9 Hz<br>$J_b(^{1}H,^{1}H)$ = 17.28 Hz
**C72**	172.89	-		
**C7A**	21.21	0.93	s	

**C8**	48.33	3.32	t	*J* (^1H,^1H) = 6.47 Hz
**C81**	22.32	1.9-2.05	m	
**C82**	32.68	2.55	m	
**C83**	171.52	-		
**C9**	175.53	-		
**OC136**<br>**OC176**<br>**OC185**<br>**OC25**<br>**OC36**<br>**OC75**<br>**OC86**	51.89, 51.93, 52.03, 52.12, 52.11, 52.39, 52.54	3.65_8, 3.66_0, 3.67, 3.69, 3.70, 3.71, 3.75	s	

Table S2. Observed signals between *H*C5, *H*C51 and *H*C8 in the NOESY experiment of **Ni-1[ClO$_4$]** (Figure S7) and calculated distances (Å) between these hydrogens in **Ni-1[ClO$_4$]** with either a *R*-, or *S*-configuration at C5. The structures of the two epimers were calculated with DFT.

	NOESY signals (experimental)	Isomer with *R*-configuration at C5 (calculated distances in Å)	Isomer with *S*-configuration at C5 (calculated distances in Å)
	*H*C8	*H*C8	*H*C8
*H*C5	Signal	3.234	2.71
*H*C51	No signal	4.63	3.15

Note: Green: Agreement between experimental and calculated data. Red: Disagreement between experimental and calculated data.

The assignment of an *R*-configuration at C5 of **Ni-1[ClO$_4$]** is based on the absence of a NOE between HC8 and HC51. This situation is in disagreement with the calculated distance in the *S*-isomer.

Electrochemistry and Analysis:

Table S3. Overview of electrodes and cells used in electrochemical studies.

Experiment / Parameter	Cyclic voltammetry / electrocatalysis	Bulk electrolysis / Bulk electrocatalysis	Spectro electrochemistry
Working electrode	GC disc	Pt mesh	Pt mesh
Counter electrode	Pt rod	GC rod	Pt wire
Reference electrode	Ag/AgCl in MeCN	Ag/AgCl in MeCN	Ag quasi
Electrolyte concentration	0.1 M	0.1 M	0.3 M
Cell	10 mL cell (one pot)	H-cell	OTTLE cell

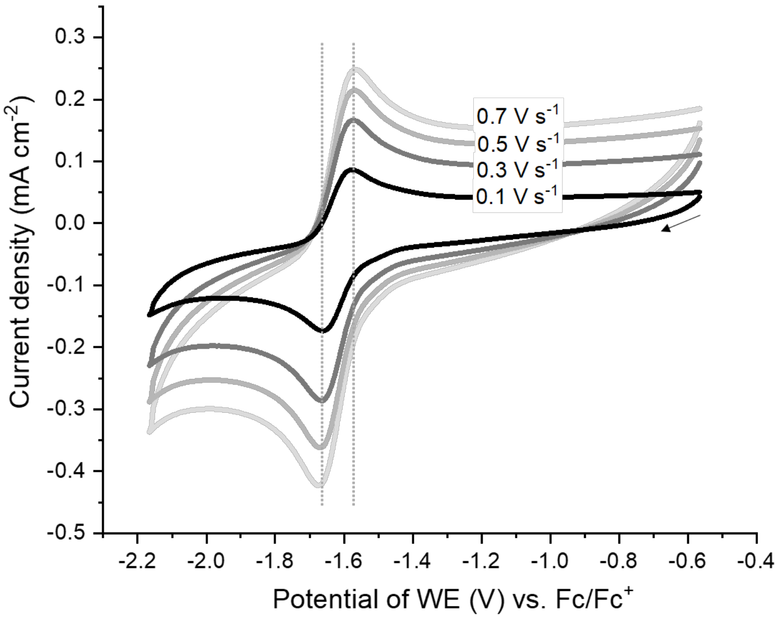

Figure S10. Cyclic voltammograms of **Ni-1[ClO₄]** (1.5 mM) at 0.1, 0.3, 0.5, 0.7 V s^{-1} taken in CH_3CN containing $TBAPF_6$ (0.1 M). CV scans were initiated at -0.56 V vs. Fc/Fc^+.

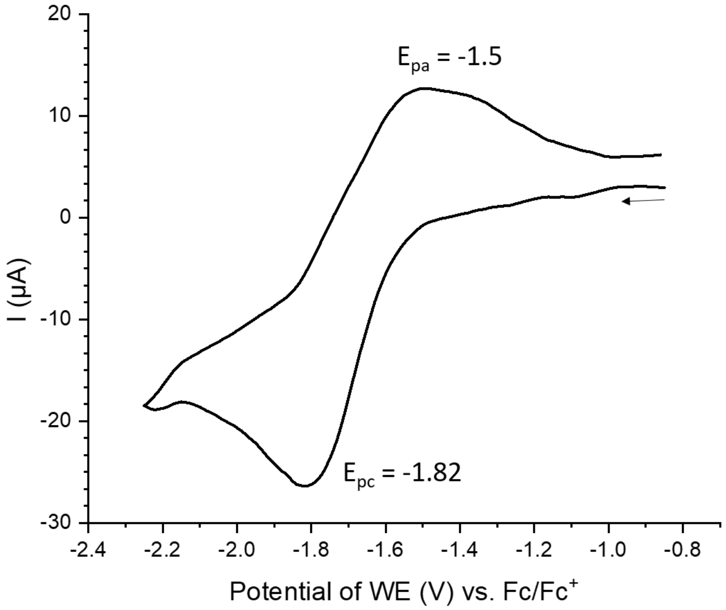

Figure S11. Cyclic voltammograms of **Ni-1[ClO₄]** (2.6 mM) at 0.1 V s^{-1} taken in CH_3CN containing $TBAPF_6$ (0.3 M) in OTTLE cell. CV scans were initiated at -0.85 V vs. Fc/Fc^+.

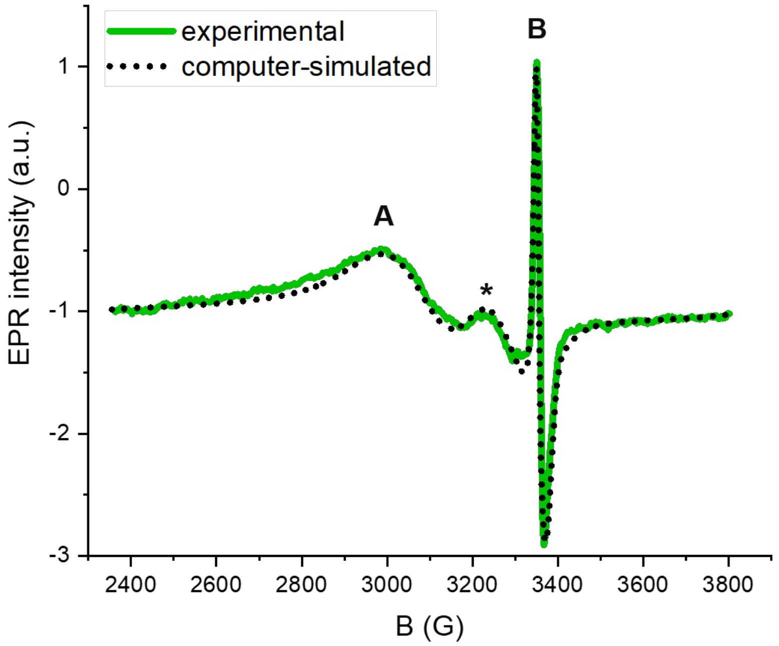

Figure S12. CW-EPR spectrum of **Ni-1** in frozen toluene/MeCN recorded at 10 K, as described in the general part. Experimental spectrum (green solid line), computer-simulated spectrum (black dotted line). They consist of metal-localized paramagnetic center (A, 95%), a ligand-centered radical (B, 5%), and resonator background (asterisk).

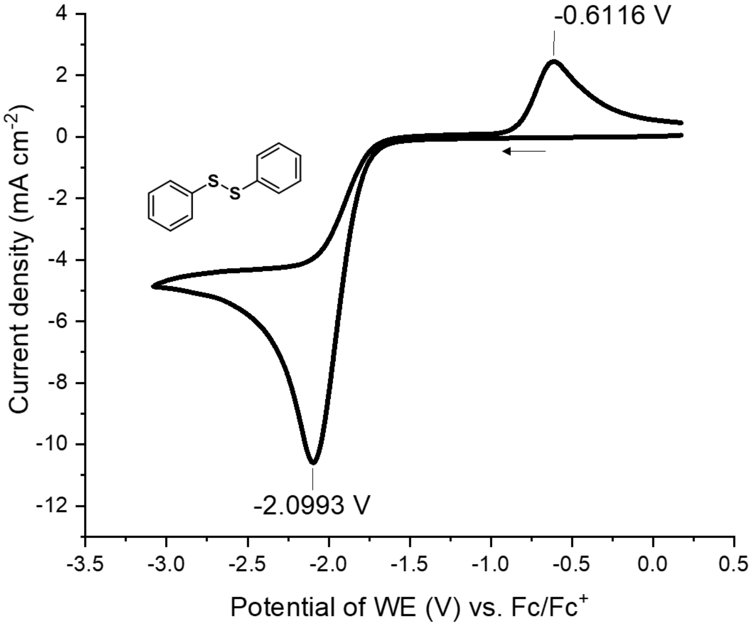

Figure S13. Cyclic voltammograms of diphenyldisulphide (**PhSSPh**) (20 mM) at 0.1 V s^{-1} taken in CH_3CN containing $TBAPF_6$ (0.1 M). CV scans were initiated at -0.526 V vs. Fc/Fc^+.

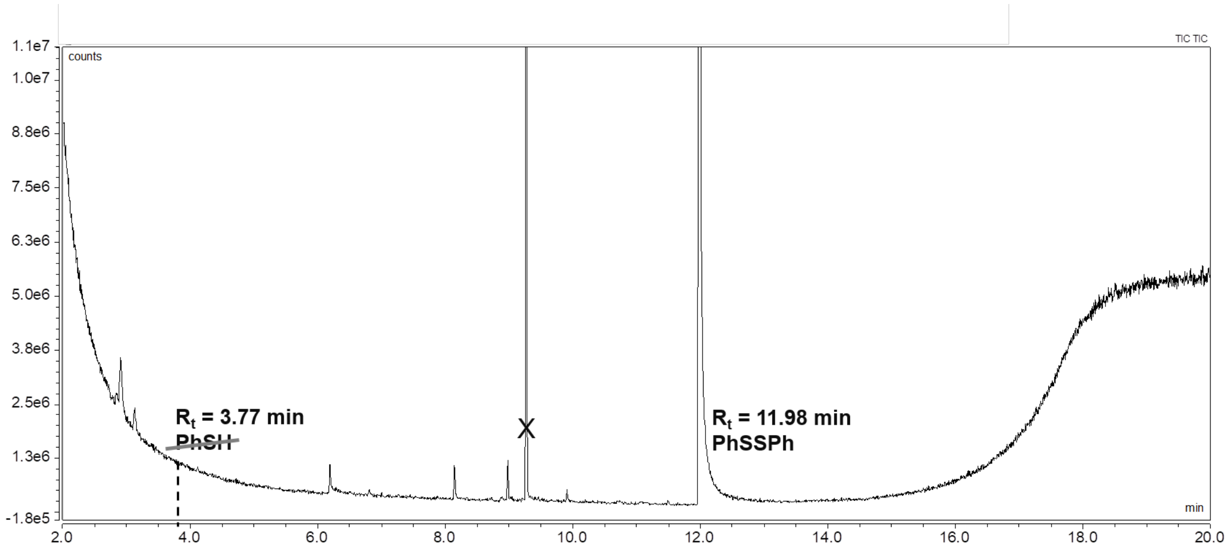

Figure S14. GC-MS chromatogram of the CPE experiment with **PhSSPh** (20 mM; at R_t = 11.98 min) in the absence of **Ni-1[ClO$_4$]** after workup and sample preparation as decribed in the experimental part [X: 2,6-Di-*tert*-butyl-4-methylphenol present as stabilizer in Et_2O]; undefined side products are not indicated]

Note: Any peak was not observed at R_t = 3.77 min indicating that no **PhSH** is formed in this experiment.

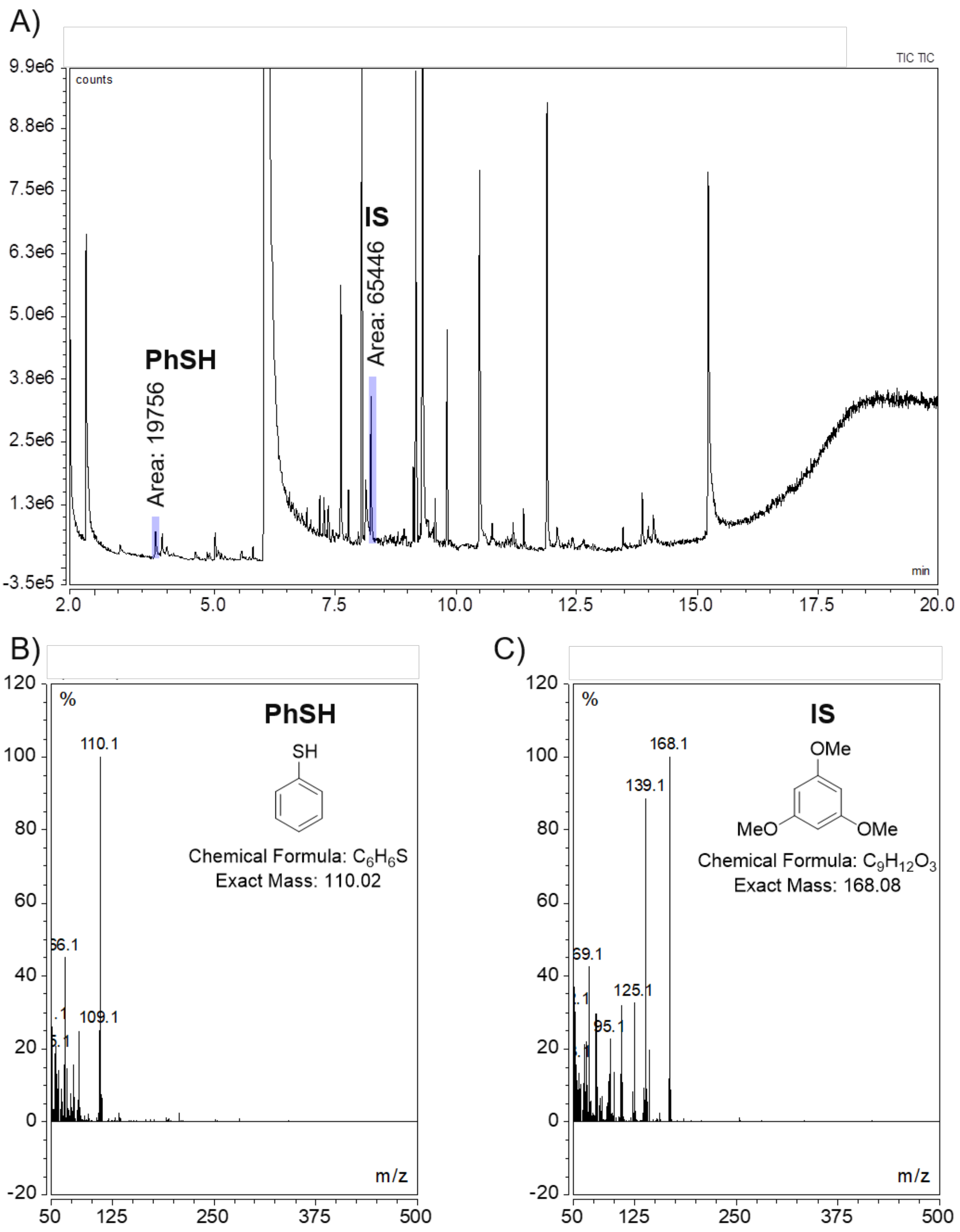

Figure S15. A. GC-MS chromatogram of the CPE experiment with **PhSSPh** (20 mM) and **Ni-1[ClO$_4$]** (1 mM) after workup and sample preparation as described in the experimental part (**PhSH**: R_t = = 3.77 min, m/z = 110.1, area = 19756; **IS**: R_t = 8.24 min, m/z =168.1, area = 65446). B. MS spectrum of **PhSH** at R_t = 3.77 min. C. MS spectrum of **IS** at 8.24 min. Ratio $Area_{PhSH}/Area_{IS}$ = 0.302. [**PhSSPh** and undefined side products are not indicated]

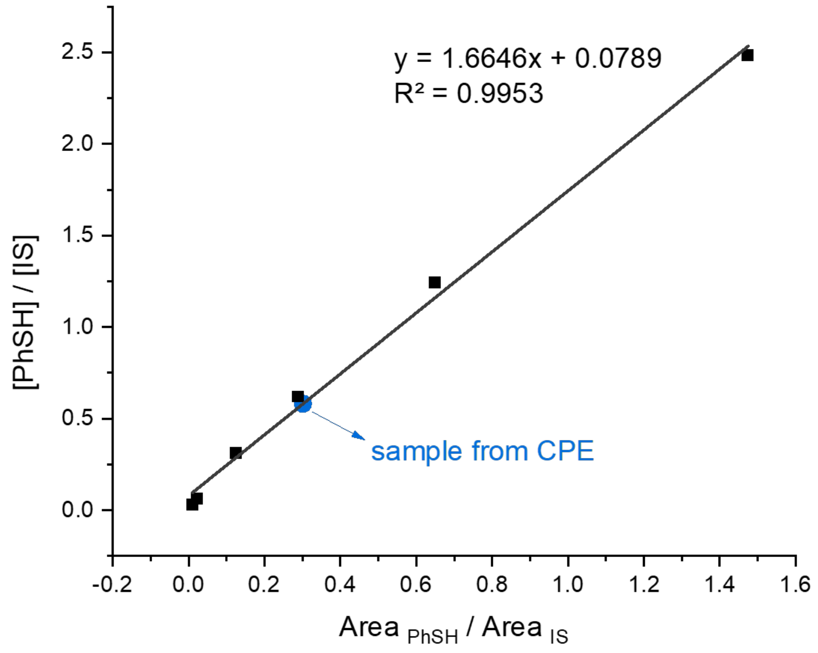

Figure S16. Calibration curve and quantification of $\mathbf{[PhSH]}_{sample}$ (blue dot) of the CPE experiment described in Figure S15.

Note:

$$[\mathbf{PhSH}]_{Sample} = ([\mathbf{IS}] \text{ x } (1.6646 \,(\text{Area}_{\mathbf{PhSH}}/\text{Area}_{\mathbf{IS}}) + 0.0789) \text{ x}16 \quad (2)$$

with $\text{Area}_{\mathbf{PhSH}}/\text{Area}_{\mathbf{IS}}$ **= 0.302** derived from Figure S15

$\mathbf{[PhSH]_{Sample}}$ = 0.552 (0.552 equiv.)

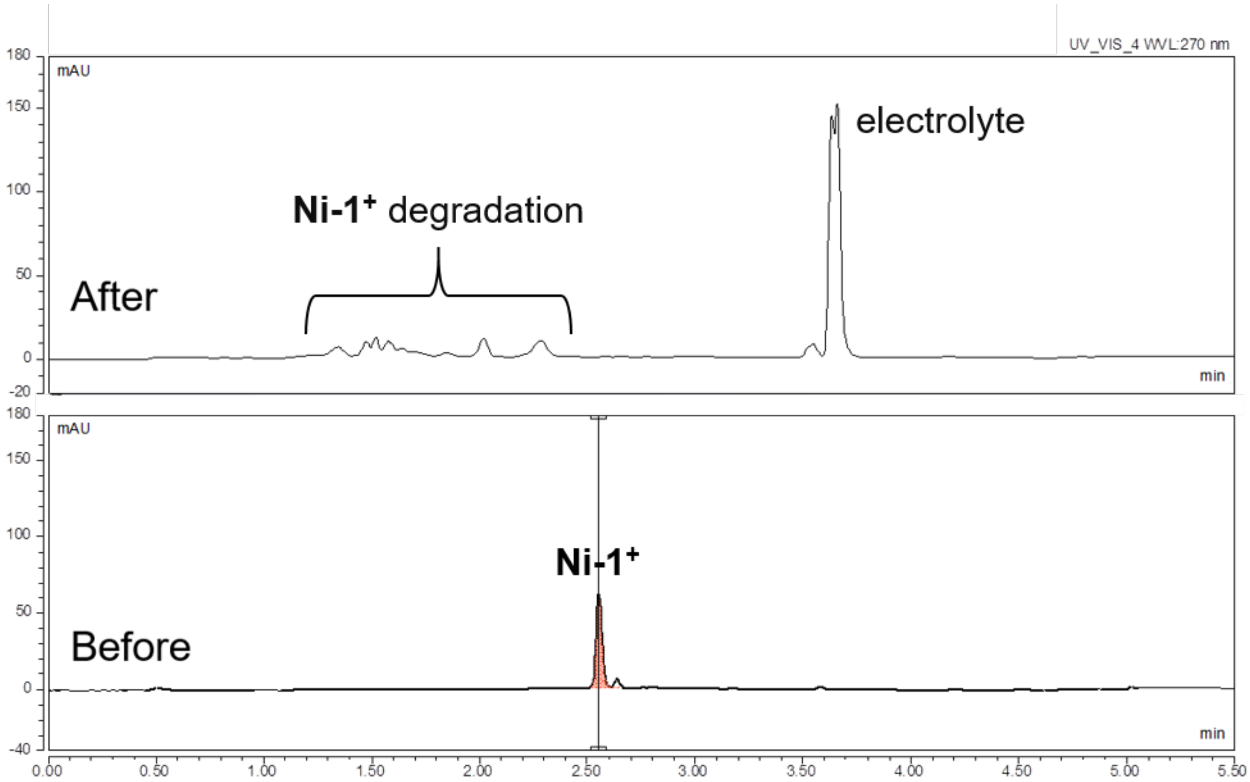

Figure S17. Chromatogram of **Ni-1[ClO$_4$]** before and after CPE (see details in experimental part). Degradation of **Ni-1[ClO$_4$]** (1 mM, R_t = 2.56 min) was observed to undefined products (R_t = 1.3–2.4 min).

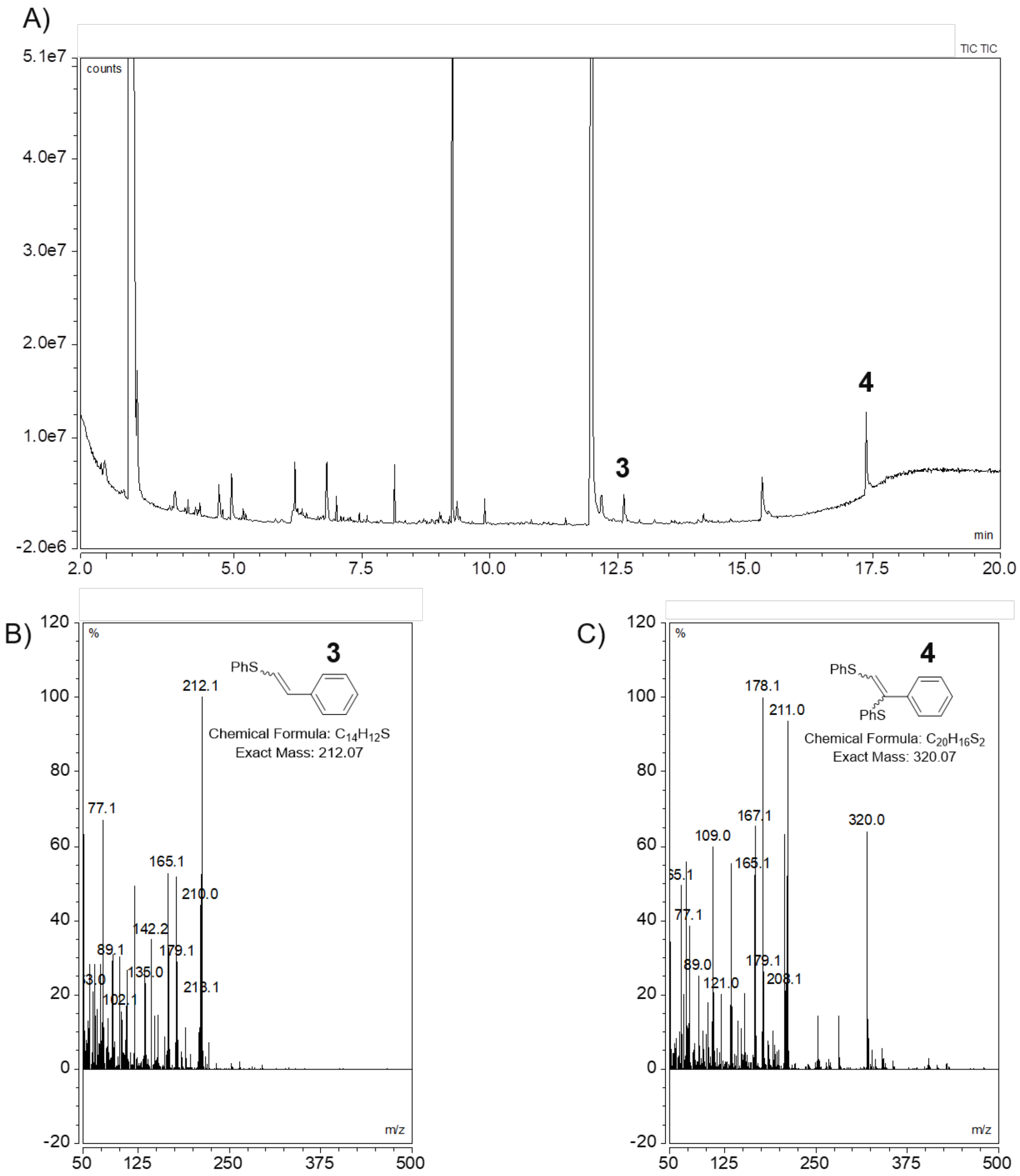

Figure S18. GC-MS of the reaction mixture (after workup) resulting from the bulk electrocatalysis of **PhSSPh** (20 mM) by **Ni-1[ClO$_4$]** (1 mM) in presence of the radical trap phenylacetylene (PA, 100 mM). A) Olefin **3** and **4** were detected on the chromatogram at R_t = 12.63 and 17.37 min respectively. B) and C) Mass spectra extracted from the GC-MS chromatogram of the trapped olefins **3** and **4**.

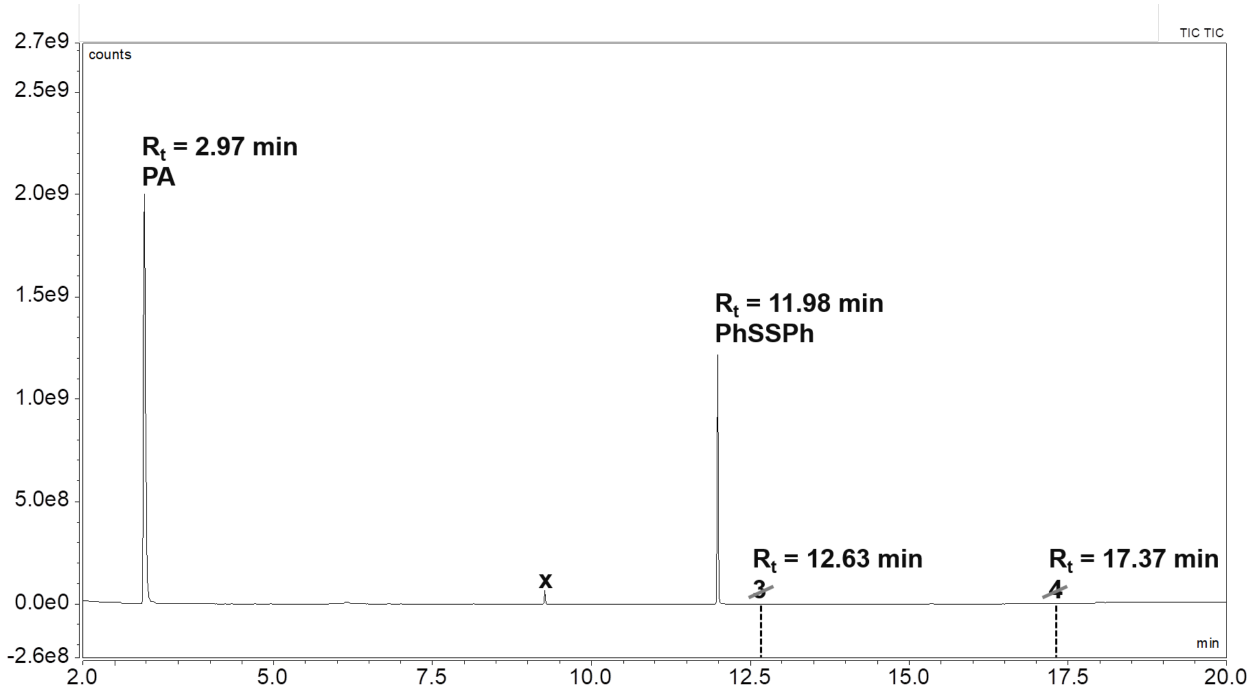

Figure S19. GC-MS chromatogram of the CPE experiment with **PhSSPh** (20 mM, at R_t = 11.98 min) with **PA** (100 mM, R_t = 2.97 min) in the absence of **Ni-1[ClO$_4$]** after workup and sample preparation as described in the experimental part. [X: 2,6-Di-tert-butyl-4-methylphenol present as stabilizer in Et_2O]

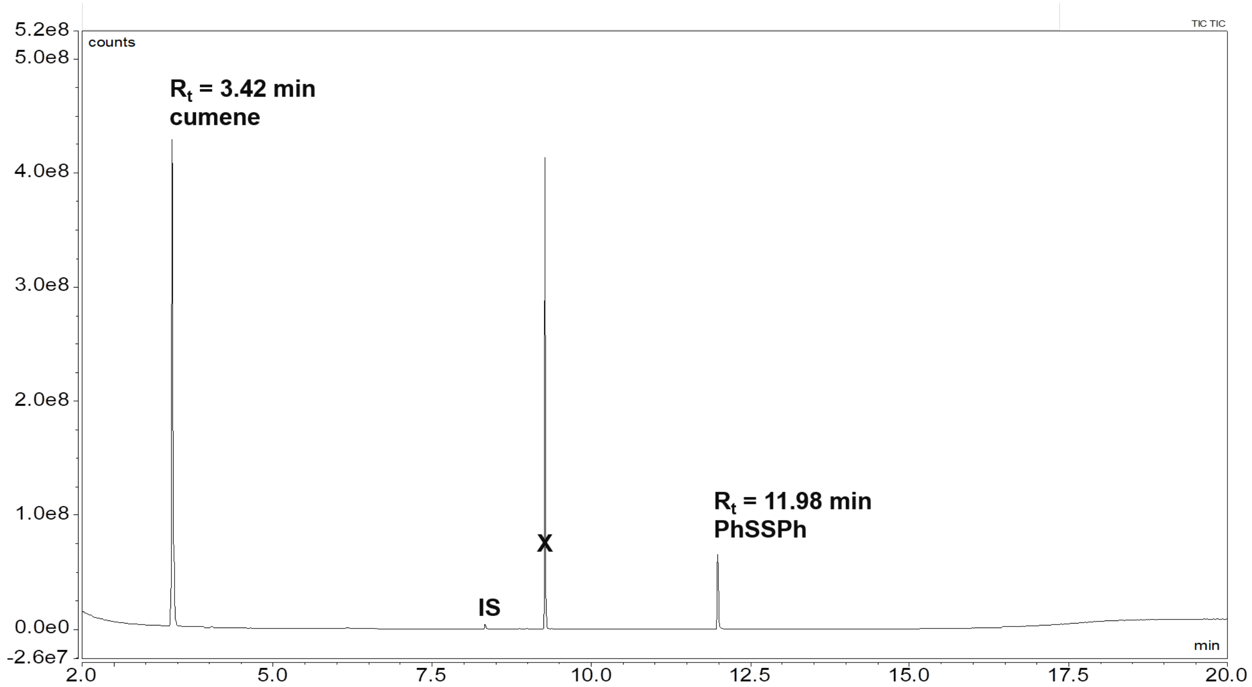

Figure S20. GC-MS of the reaction mixture (after workup) resulting from the bulk electrocatalysis of **PhSSPh** (20 mM, at R_t = 11.98 min) by **Ni-1[ClO$_4$]** (1 mM) in presence of **cumene** (100 mM, at R_t = 3.42 min). [X: 2,6-Di-tert-butyl-4-methylphenol present as stabilizer in Et_2O; **IS**: internal standard]

Computational Data:

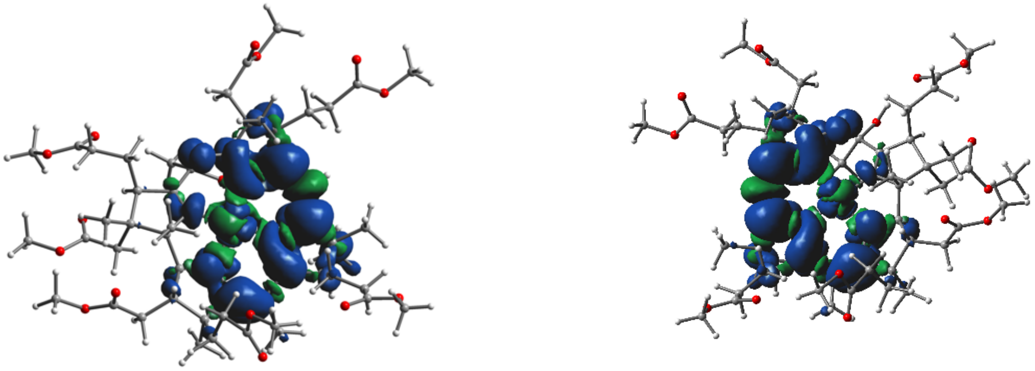

Figure S21. Optimized structures of **Ni-1[ClO$_4$]** with an *R*-configuration at C5 (left) and a *S*-configuration at C5 (right) showing the spin density.

Table S4. Cartesian coordinates of the DFT optimized ground-state structure of **Ni-1$^+$**.

1 1

C	2.39701248	-0.68003536	0.34696533
C	3.87030277	-0.04707436	0.26941277
C	3.59211380	1.46128226	-0.09227005
C	2.17074462	1.45749421	-0.56570893
C	1.63375013	2.62015118	-1.39492513
C	-0.45309315	2.06302473	1.39506705
C	-1.40695937	3.20943387	1.77111213
C	-2.21205135	3.39666788	0.45421752
C	-2.08963180	2.01656744	-0.15981572
C	-3.01988731	1.40976980	-1.00369494
C	-2.95366501	0.06055351	-1.36068777
C	-4.05184956	-0.67595170	-2.11969709
C	-3.69573368	-2.15348766	-1.76893311
C	-2.20446734	-2.05883394	-1.49773255
C	-1.32475656	-3.12959064	-1.53749302

C	0.05376598	-2.95965100	-1.15078287
C	1.10148126	-4.07615600	-1.02128531
C	2.23046148	-3.34047473	-0.20829620
C	2.04813347	-1.86578334	-0.62042340
N	1.52401427	0.39218859	-0.23207161
N	-0.96092456	1.39484211	0.25698338
N	-1.89873128	-0.76251981	-1.11235578
N	0.57739719	-1.78473124	-0.85269050
C	1.92753002	-1.00849968	1.77171462
C	4.64983781	-0.23877010	1.58326466
C	3.75144842	2.55808608	1.00026335
C	5.19820508	3.02803941	1.20097201
C	5.78277447	3.63406315	-0.06237243
O	5.13314003	4.13425789	-0.96838653
O	7.12957190	3.56245742	-0.06518876
C	7.78590546	4.04635187	-1.25977212
O	0.52567216	1.73344755	2.04343955
C	-2.29754944	2.70216891	2.93090339
C	-0.57643987	4.44782903	2.19826493
C	-3.62201487	3.99903022	0.57296650
C	-4.07022950	4.75114805	-0.68941868
C	-5.31385723	5.58402687	-0.44383281
O	-5.56910397	6.17047570	0.59787851
O	-6.10617395	5.63235377	-1.53296081
C	-7.29085115	6.45632376	-1.41940406
C	-5.47385751	-0.22186483	-1.75734408
C	-3.79993592	-0.44505076	-3.63644702
C	-4.35881428	-2.76075734	-0.50020589
C	-1.85225629	-4.48527040	-1.97337838

C	1.59045163	-4.49800277	-2.42930260
C	0.59856238	-5.30486291	-0.21799419
C	-0.12899490	-4.94709213	1.09051337
C	-1.24950577	-5.90826526	1.44961422
O	-1.74729797	-6.73883435	0.70465292
O	-1.67961572	-5.69013723	2.70766400
C	-2.83678399	-6.45630127	3.12752030
C	3.60915360	-3.98284932	-0.39660592
C	4.56497436	-3.75088327	0.75778541
O	4.23871812	-3.62450086	1.92866986
O	5.84121252	-3.73351797	0.33022784
C	6.86686196	-3.56445429	1.33966171
H	-3.91131365	-2.80940839	-2.62776153
H	1.96986425	-3.42167528	0.85924864
H	2.52773055	-1.67324038	-1.59570743
H	2.55124161	-1.80595685	2.20248110
H	0.88041369	-1.35715524	1.74201110
H	5.67522147	0.14834710	1.49078922
H	4.16793303	0.27861020	2.42482431
H	4.72305917	-1.30382477	1.84510591
H	3.31742667	2.21591382	1.95288181
H	3.14937357	3.42722464	0.68642877
H	5.86731027	2.22781026	1.55188251
H	5.22182403	3.81373325	1.97957201
H	8.86232972	3.98038700	-1.05207845
H	7.51336585	3.39979490	-2.10910755
H	7.49020053	5.08704884	-1.46861223
H	-2.96011063	3.50611124	3.28947872
H	-2.91592648	1.84611907	2.61020155

H	-1.65915153	2.37955580	3.77020221
H	0.20400509	4.63197751	1.44668484
H	-0.10066755	4.23697513	3.16872942
H	-3.64136811	4.71691289	1.40949305
H	-4.35640808	3.21592658	0.83132705
H	-4.24406168	4.08621003	-1.55005033
H	-3.27583947	5.45995846	-0.99419448
H	-3.89674357	0.62476915	-3.88728433
H	-2.78956693	-0.78312090	-3.92567425
H	-4.54120616	-1.01330918	-4.22438423
H	-1.10763802	-5.04165017	-2.56022170
H	-2.74074196	-4.37186606	-2.61099823
H	-2.13843060	-5.12290416	-1.11855495
H	0.75570140	-4.86974058	-3.04325178
H	2.33202612	-5.31077027	-2.35474812
H	2.05251810	-3.65158882	-2.96471451
H	1.47545080	-5.94004817	0.00405050
H	-0.06264392	-5.92589929	-0.83969876
H	-0.61724562	-3.95464879	1.01423982
H	0.55265002	-4.87093415	1.95385343
H	-2.97729420	-6.21381082	4.18954714
H	-3.71747331	-6.15349726	2.53828738
H	-2.64901884	-7.53412055	2.99616848
H	4.08877642	-3.70216663	-1.34681714
H	3.48351703	-5.08226882	-0.43159392
H	7.81946684	-3.70465303	0.81263155
H	6.81124088	-2.54905647	1.76129165
H	6.74142869	-4.31247946	2.13808656
H	-3.70092075	-3.56104428	-0.12272404

H	-4.39687264	-2.00046578	0.30113254
H	-7.79607117	6.38176549	-2.39144581
H	-7.94015238	6.07962550	-0.61233779
H	-7.00951502	7.50020743	-1.20436852
H	-3.90501497	1.97158692	-1.30106537
H	-6.21320486	-0.83713057	-2.29642931
H	-5.67006839	-0.29378969	-0.67519001
H	-5.63588883	0.82570861	-2.06321331
C	-1.44959831	5.67500017	2.34283989
O	-2.11620125	5.96353570	3.32451634
O	-1.45140804	6.40959148	1.20480244
C	-2.32216867	7.56789550	1.19989758
H	-2.12542197	8.07671715	0.24677866
H	-3.37586572	7.24755048	1.25728968
H	-2.08523878	8.22518714	2.05155226
H	-1.59575068	4.05151334	-0.19376265
H	1.96338298	-0.11565243	2.40692002
C	4.69183582	-0.65437931	-0.91743915
H	4.14400248	-0.51649183	-1.86300130
H	4.86755954	-1.72513890	-0.75430131
C	6.03948772	0.01751474	-1.10102049
O	6.22853365	1.04057706	-1.74401297
O	7.02159513	-0.62981727	-0.44235298
C	8.33037731	-0.00794206	-0.48442451
H	8.98096571	-0.65921499	0.11363045
H	8.68679983	0.05390378	-1.52505363
H	8.27560905	1.00414909	-0.05236535
H	4.23428341	1.76708978	-0.93605782
Ni	-0.23266877	-0.16887939	-0.50432214

C	-5.74146001	-3.38597585	-0.71847140
H	-6.54453792	-2.64522533	-0.85096544
H	-5.73393215	-4.00558094	-1.63666074
C	-6.11625276	-4.31999795	0.42030337
O	-5.32458074	-4.90585657	1.14241609
O	-7.45356220	-4.45619646	0.52533118
C	-7.91854586	-5.37626649	1.54081701
H	-9.01470224	-5.35390308	1.47942719
H	-7.53782638	-6.39108181	1.33957059
H	-7.57708858	-5.04921066	2.53648365
H	2.54083773	3.12605043	-1.77610788
O	0.93859779	3.53537989	-0.52734957
H	0.94680255	4.40748946	-0.96436660
C	0.77282741	2.17989643	-2.58211837
H	1.31333021	1.44557787	-3.20297865
H	-0.17153750	1.72596762	-2.23871883
H	0.53374136	3.05792154	-3.20520428

Table S5. Cartesian coordinates of the DFT optimized ground-state structure of **Ni-1**.

C	2.53595670	-0.42147548	0.29423536
C	4.03919465	0.16708067	0.28012797
C	3.84224523	1.64825732	-0.22538787
C	2.39700318	1.69209970	-0.64078363
C	1.83276499	2.93873446	-1.30362061
C	-0.68830872	2.32224318	1.27676393
C	-1.79520615	3.30823300	1.70831132
C	-2.71057359	3.28516189	0.45139599
C	-2.38981298	1.90949191	-0.11265035
C	-3.23203693	1.16053003	-0.92152989

C	-2.96697467	-0.13439607	-1.40519036
C	-3.93089174	-0.92905289	-2.28772494
C	-3.45466983	-2.37721417	-1.95080999
C	-1.98296060	-2.13425515	-1.62685440
C	-1.00010679	-3.12295831	-1.65767337
C	0.34076325	-2.86800745	-1.19908420
C	1.38739118	-3.94773178	-0.86973500
C	2.39750786	-3.11400045	-0.00486938
C	2.24750169	-1.68905631	-0.59092233
N	1.70649937	0.64231475	-0.35032205
N	-1.13864494	1.49097567	0.27806675
N	-1.83044560	-0.83817682	-1.17159115
N	0.81660982	-1.64182289	-1.00024788
C	1.96411678	-0.63221287	1.70965845
C	4.69138703	0.07464924	1.67251170
C	4.20039072	2.82251572	0.72695655
C	5.69586721	3.15564645	0.78474543
C	6.22599793	3.69099348	-0.53265076
O	5.54535481	4.12666256	-1.44810535
O	7.57541907	3.64372989	-0.56910029
C	8.18813706	4.03968655	-1.81620533
O	0.44253525	2.31157015	1.79597853
C	-2.48337972	2.73150745	2.96553713
C	-1.16050007	4.69714829	1.98442547
C	-4.19527809	3.62939334	0.67260875
C	-4.87162520	4.25916816	-0.55505036
C	-6.23047493	4.84160290	-0.21874724
O	-6.51113943	5.40809867	0.82771061
O	-7.10790002	4.68760158	-1.23162943

C	-8.42003391	5.26164331	-1.02483361
C	-5.41454764	-0.62707657	-2.02914481
C	-3.59604669	-0.62298821	-3.77219864
C	-4.11565584	-3.05612833	-0.72128730
C	-1.40901443	-4.51460654	-2.11830725
C	2.05148351	-4.45611927	-2.17499004
C	0.83781351	-5.14121233	-0.04625565
C	-0.02904087	-4.73315325	1.15674418
C	-1.09825548	-5.75085503	1.50829819
O	-1.55603731	-6.59608688	0.75331894
O	-1.54013583	-5.57395975	2.77034879
C	-2.65386171	-6.40854440	3.17534493
C	3.78853880	-3.76021703	0.04520544
C	4.60181180	-3.43429623	1.28059737
O	4.15002819	-3.27099802	2.40405378
O	5.91836656	-3.38531639	0.99075935
C	6.82443009	-3.13615684	2.09126983
H	-3.57819354	-3.03407330	-2.82817218
H	2.00182571	-3.09193553	1.02351678
H	2.83701726	-1.60075719	-1.52071521
H	2.55429137	-1.36696281	2.27755038
H	0.92756491	-1.00609184	1.62064183
H	5.72924311	0.43612194	1.65008922
H	4.14330408	0.66398791	2.42107617
H	4.71346283	-0.96486822	2.02724008
H	3.82059018	2.62189841	1.74152300
H	3.66719776	3.71962006	0.37381771
H	6.31863888	2.29781532	1.08564354
H	5.87508140	3.93764100	1.54666269

H	9.27146666	4.00079919	-1.63947209
H	7.89805204	3.32845159	-2.60646868
H	7.87397490	5.05805882	-2.09654742
H	-3.25074125	3.42271334	3.35003500
H	-2.95919663	1.76212412	2.73692904
H	-1.73331640	2.57509172	3.75944279
H	-0.53630312	4.98495562	1.12360814
H	-0.52501805	4.62728460	2.88132713
H	-4.28597955	4.35151891	1.50168263
H	-4.75204814	2.73027393	0.99135633
H	-4.97631076	3.55688301	-1.39691964
H	-4.25262728	5.10201729	-0.91960157
H	-3.77960849	0.44174780	-3.99793551
H	-2.53696584	-0.84836222	-3.98963381
H	-4.22664749	-1.23670958	-4.44003262
H	-0.58319991	-5.03967407	-2.61966623
H	-2.23375763	-4.45841667	-2.84514678
H	-1.74921390	-5.15981720	-1.28756188
H	1.30421564	-4.89855537	-2.85190784
H	2.80062511	-5.23645096	-1.95829184
H	2.55095952	-3.63499250	-2.71691679
H	1.69704677	-5.74400414	0.30386468
H	0.25433898	-5.81213407	-0.69381126
H	-0.58330212	-3.79978900	0.92836177
H	0.56312179	-4.51716733	2.06093334
H	-2.82930555	-6.16807202	4.23274065
H	-3.54052296	-6.16973740	2.56645487
H	-2.39468388	-7.47336714	3.05766979
H	4.38363907	-3.56622517	-0.86008338

H	3.66066661	-4.85957903	0.09455895
H	7.83202712	-3.23219419	1.66635417
H	6.67181019	-2.11790569	2.48151976
H	6.66116827	-3.87282617	2.89377458
H	-3.40485499	-3.79323520	-0.31221982
H	-4.26422519	-2.30651322	0.07802358
H	-8.98501873	5.04461276	-1.94109274
H	-8.90408493	4.79899053	-0.14926951
H	-8.33956722	6.34927565	-0.86433436
H	-4.19285910	1.59815633	-1.19717690
H	-6.05154047	-1.28835733	-2.64121728
H	-5.68823198	-0.75224375	-0.96803910
H	-5.65397463	0.41146817	-2.31696310
C	-2.21913145	5.75219951	2.21678305
O	-2.80635909	5.95177267	3.26921573
O	-2.49154487	6.43522303	1.07849837
C	-3.55907977	7.41026460	1.16383558
H	-3.56474352	7.92283690	0.19246034
H	-4.52187380	6.90062003	1.33602588
H	-3.36161657	8.12211177	1.98117016
H	-2.28688274	4.02904222	-0.25473503
H	1.92230341	0.32383468	2.24953155
C	4.94158076	-0.56768559	-0.76637295
H	4.45995504	-0.54206366	-1.75573186
H	5.10138930	-1.61145424	-0.46871947
C	6.30150232	0.07498074	-0.95147714
O	6.55469305	0.95067448	-1.76731709
O	7.22320134	-0.41852231	-0.09665655
C	8.54328987	0.17490792	-0.17452834

H	9.12853086	-0.31359231	0.61559909
H	8.98560384	-0.01970653	-1.16498864
H	8.47925429	1.26239855	-0.00969598
H	4.44165934	1.80864216	-1.14059807
Ni	-0.14068888	-0.02784538	-0.58768398
C	-5.42356684	-3.80239360	-1.00631055
H	-6.26101375	-3.13742458	-1.26748192
H	-5.28827326	-4.48154474	-1.87134738
C	-5.84366790	-4.67431341	0.16317076
O	-5.09867689	-5.10556009	1.02946458
O	-7.16531644	-4.94761104	0.12475957
C	-7.66186330	-5.81893717	1.16693144
H	-8.73960066	-5.91823390	0.98058315
H	-7.16587642	-6.80214808	1.11243812
H	-7.47758912	-5.37270809	2.15796491
H	2.68927338	3.43743835	-1.79584874
O	1.38449814	3.85447489	-0.28368295
H	1.14708682	3.32147456	0.51345457
C	0.75300098	2.67500193	-2.35417315
H	1.15639610	2.05232278	-3.17153282
H	-0.11445582	2.15408491	-1.91461083
H	0.41704158	3.63760574	-2.77479538

References:

[S1] L. Werthemann, Untersuchungen an Kobalt (II) - Und Kobalt (III)-Komplexen Des Cobyrinsäure-Heptamethylesters, Dissertation ETH Zürich, **1968**.

[S2] S. M. Chemaly, K. L. Brown, M. A. Fernandes, O. Q. Munro, C. Grimmer, H. M. Marques, *Inorg. Chem.* **2011**, *50*, 8700–8718.

[S3] B. Kräutler, R. Stepánek, *Helv. Chim. Acta* **1985**, *68*, 1079–1088.

[S4] C. Brenig, L. Mosberger, O. Blacque, R. Kissner, F. Zelder, *Chem. Commun.* **2021**, *57*, 7260–7263.

[S5] S. Stoll, A. Schweiger, *J. Magn. Reson.* **2006**, *178*, 42–55.

[S6] Gaussian 09, Revision D.01, M. J. Frisch, G. W. Trucks, H. B. Schlegel, G. E. Scuseria, M. A. Robb, J. R. Cheeseman, G. Scalmani, V. Barone, B. Mennucci, G. A. Petersson, H. Nakatsuji, M. Caricato, X. Li, H. P. Hratchian, A. F. Izmaylov, J. Bloino, G. Zheng, J. L. Sonnenberg, M. Hada, M. Ehara, K. Toyota, R. Fukuda, J. Hasegawa, M. Ishida, T. Nakajima, Y. Honda, O. Kitao, H. Nakai, T. Vreven, J. A. Montgomery, Jr., J. E. Peralta, F. Ogliaro, M. Bearpark, J. J. Heyd, E. Brothers, K. N. Kudin, V. N. Staroverov, T. Keith, R. Kobayashi, J. Normand, K. Raghavachari, A. Rendell, J. C. Burant, S. S. Iyengar, J. Tomasi, M. Cossi, N. Rega, J. M. Millam, M. Klene, J. E. Knox, J. B. Cross, V. Bakken, C. Adamo, J. Jaramillo, R. Gomperts, R. E. Stratmann, O. Yazyev, A. J. Austin, R. Cammi, C. Pomelli, J. W. Ochterski, R. L. Martin, K. Morokuma, V. G. Zakrzewski, G. A. Voth, P. Salvador, J. J. Dannenberg, S. Dapprich, A. D. Daniels, O. Farkas, J. B. Foresman, J. V. Ortiz, J. Cioslowski, and D. J. Fox, Gaussian, Inc., Wallingford CT, **2013**.

[S7] J. M. Tao, J. P. Perdew, V. N. Staroverov, and G. E. Scuseria, *Phys. Rev. Lett.*, **2003**, *91,* 146401.

[S8] T. H. Jr Dunning, P. Hay, *J. Modern Theor. Chem.* Plen. New York, 1976; Vol. 3.

[S9] F. Weigend and R. Ahlrichs, *Phys. Chem. Chem. Phys*., **2005**, *7*, 3297-3305.

[S10] F. Weigend, *Phys. Chem. Chem. Phys.*, **2006**, *8*, 1057-1065.

[S11] M. Cossi, N. Rega, G. Scalmani, V. Barone, *J. Comput. Chem.* **2003**, *24*, 669-681.

[S12] R. Bauernschmitt, R. Ahlrichs, *Chem. Phys. Lett.* **1996**, *256*, 454-464.
